# Chiral Cyclobutane-Containing Cell-Penetrating Peptides as Selective Vectors for Anti-*Leishmania* Drug Delivery Systems

**DOI:** 10.3390/ijms21207502

**Published:** 2020-10-12

**Authors:** Ona Illa, José-Antonio Olivares, Nerea Gaztelumendi, Laura Martínez-Castro, Jimena Ospina, María-Ángeles Abengozar, Giuseppe Sciortino, Jean-Didier Maréchal, Carme Nogués, Míriam Royo, Luis Rivas, Rosa M. Ortuño

**Affiliations:** 1 Departament de Química, Universitat Autònoma de Barcelona, 08193 Cerdanyola del Vallès, Spain; ona.illa@uab.cat (O.I.); joseanolivares1@gmail.com (J.-A.O.); laura.martinezcas@e-campus.uab.cat (L.M.-C.); jimenaabcn@gmail.com (J.O.); Giuseppe.Sciortino@uab.cat (G.S.); JeanDidier.Marechal@uab.cat (J.-D.M.); 2 Departament de Biologia Cel·lular, Fisiologia i Immunologia, Universitat Autònoma de Barcelona, 08193 Cerdanyola del Vallès, Spain; negazte@gmail.com; 3 Centro de Investigaciones Biológicas Margarita Salas, CSIC, c/Ramiro de Maeztu, 9, 28040 Madrid, Spain; marabi66@hotmail.com; 4 Institut de Química Avançada de Catalunya (IQAC-CSIC), c/Jordi Girona, 18–26, 08034 Barcelona, Spain; miriam.royo@iqac.csic.es; 5 Centro de Investigación Biomédica en Red Bioingeniería, Biomateriales y Nanomedicina (CIBER-BBN), c/Jordi Girona, 18–26, 08034 Barcelona, Spain

**Keywords:** unnatural γ-amino acids, foldamers, selective cell-penetrating peptides, anti-*Leishmania* drug delivery vectors

## Abstract

Two series of new hybrid γ/γ-peptides, γ-CC and γ-CT, formed by (1*S*,2*R*)-3-amino-2,2,dimethylcyclobutane-1-carboxylic acid joined in alternation to a *N*^α^-functionalized *cis*- or *trans*-γ-amino-l-proline derivative, respectively, have been synthesized and evaluated as cell penetrating peptides (CPP) and as selective vectors for anti-*Leishmania* drug delivery systems (DDS). They lacked cytotoxicity on the tumoral human cell line HeLa with a moderate cell-uptake on these cells. In contrast, both γ-CC and γ-CT tetradecamers were microbicidal on the protozoan parasite *Leishmania* beyond 25 μM, with significant intracellular accumulation. They were conjugated to fluorescent doxorubicin (Dox) as a standard drug showing toxicity beyond 1 μM, while free Dox was not toxic. Intracellular accumulation was 2.5 higher than with Dox-TAT conjugate (TAT = transactivator of transcription, taken as a standard CPP). The conformational structure of the conjugates was approached both by circular dichroism spectroscopy and molecular dynamics simulations. Altogether, computational calculations predict that the drug-γ-peptide conjugates adopt conformations that bury the Dox moiety into a cavity of the folded peptide, while the positively charged guanidinium groups face the solvent. The favorable charge/hydrophobicity balance in these CPP improves the solubility of Dox in aqueous media, as well as translocation across cell membranes, making them promising candidates for DDS.

## 1. Introduction

Novel drug delivery systems (DDS) have been developed to achieve a more effective and specific delivery of the drugs to the target organ or cell. One of the major advantages of this approach is to avoid, or at least decrease, the side-effects associated with conventional drugs [1]. In this regard, DDS, together with imaging techniques, are key tools in theranostics or personalized medicine [2,3].

Cell penetrating peptides (CPP) [4,5,6] are potential carriers for DDS. They consist of 5–30 amino acid-long peptides which are capable of transporting the cargo molecule into the intracellular space in the absence of a cognate transporter. At a higher specificity level, the inclusion of an import motif in the CPP may even make it possible to direct the cargo into specific organelles. Either covalent or noncovalent bonding between the cargo molecule and the CPP underlies their use as DDS [7,8,9]. CPP possess appealing features such as good biocompatibility, the potential to fine tune their stability and solubility in biological environments, and potential to create new multifunctional DDS [10]. As such, CPP were implemented as a new tool in the therapeutics of a variety of diseases, with special relevance for cancer [11].

Optimization of the performance of CPP aims to improve both their proteolytic resilience and cell uptake, as well as to achieve minimal toxicity. Endosomal escape ability is also an important characteristic of optimal CPP. Imperviousness to peptidases can be tackled by the inclusion of non-natural or stereochemically modified amino acids, as well as of peptide bond surrogates, in the peptide sequence [12]. Otherwise, this can also be addressed by the introduction of conformational constraints to obtain a more stable secondary structure in the CPP, which is sometimes associated with better cell uptake [12]. For cationic CPP, uptake and toxicity are highly dependent on the number and spatial distribution throughout their sequence. With these two goals in mind, CPP were designed with the inclusion of cyclic amino acids, mainly proline and γ-aminoproline [13,14], helical peptide foldamers [15,16,17], and cyclic peptide backbones [18,19,20].

**Figure 1:**
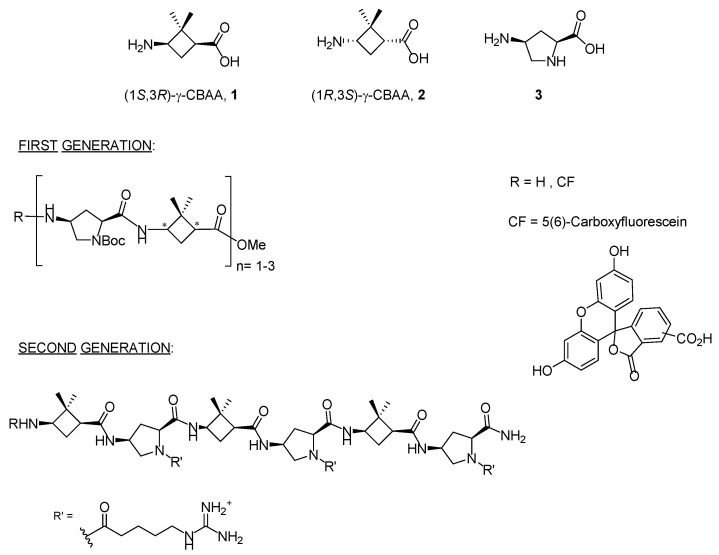
Monomers used in the first and second generations of hybrid cyclobutane/proline γ/γ-peptides labelled with 5(6)-carboxyfluorescein (CF).

In recent years, two diastereomeric series of short cell-penetrating hybrid γ/γ-peptides were efficiently prepared through convergent synthesis in solution. These peptides were formed by repetition of a dimeric unit constituted by *cis*-γ-amino-l-proline, **3**, combined with a protected derivative of either (1*S*,3*R*)- or (1*R*,3*S*)-3-amino-2,2-dimethylcyclobutane-1-carboxylic acid, namely (1*S*,3*R*)-γ-CBAA, **1**, or (1*R*,3*S*)-γ-CBAA, **2**, (Figure 1). By high-resolution NMR, these peptides displayed very rigid and compact structures due to the intra- and inter-residue hydrogen-bonded ring formation [21].

Their uptake by HeLa cells increased with the length of the peptide; a five-carbon spacer between the proline *N*^α^ atom and the terminal guanidinium group of the side chain was optimal for cell uptake, while the stereochemistry of the γ-CBAA was largely irrelevant. A good polar-hydrophobicity balance was achieved by the alternation of the guanidinium groups and the hydrophobicity of the (*gem*-dimethyl)cyclobutane ring [21,22]. These cyclobutane-containing CPP showed lower toxicity but similar cell uptake to those made exclusively of γ-amino proline residues [23], likely due to their halved number of guanidinium groups compared with the γ-aminoproline peptides of the same length.

Nowadays, the therapeutic use of CPP-based DDS is mainly focused on cancer, running far beyond examples involving infectious diseases and their causative agents [24]. Nevertheless, infective bacteria, fungi and protozoa are an appealing test for CPP. The cell membrane of these pathogens shows an external anionic hemilayer, in contrast to the zwitterionic one of mammalian cells, privileging interactions of cationic CPP with such pathogens [25]. Furthermore, pathogenic organisms are frequently endowed with a high proteolytic armamentarium as part of their virulence program [26].

A case in point is the human protozoan parasite *Leishmania*, the causative agent for the wide clinical spectrum of leishmaniasis, a disease with a significant impact on global human health [27]. There is an urgent need for new chemotherapy alternatives for leishmaniasis [28,29,30] to reduce the side effects associated with current chemotherapy, despite the existence of some drugs such as paromomycin [31] or miltefosine [32]. Furthermore, *Leishmania* is a challenging organism for membrane active peptides [33], including CPP. All the endocytic traffic, a common pathway for CPP uptake, is carried out through the flagellar pocket [34], a specialized region accounting for only 5% of the total surface of the parasite [35]. In addition, the promastigote, responsible for the primary infection in the mammalian host, displays a highly anionic glycocalyx [36], and its main plasma membrane protein is leishmaniolysin, a Zn^2+^-metalloprotease of broad substrate specificity [37]. The aflagellated amastigote is the pathological form of the parasite in vertebrates, dwelling in the parasitophorus vacuole of the macrophage, a strong proteolytic and nutrient-demanding environment.

There are few examples of the use of CPP in the treatment of *Leishmania* [38,39,40]. Even so, the use of CPP as DDS on *Leishmania* parasites has been reported for the delivery of cargoes which are noncovalently complexed with CPP [41,42,43], or as covalent conjugates [44,45,46,47,48].

The anthraquinone doxorubicin (Dox) is broadly used in cancer chemotherapy, and its biological activity is preserved once conjugated to CPP through its amino group [11,49]. As doxorubicin also has leishmanicidal activity [50,51,52] and is a fluorescent compound, it is an excellent molecular beacon to test the functional performance of CPP as functional DDS, but also for an easy track down of intracellular fate and the accumulation of the conjugate.

**Chart 1:**
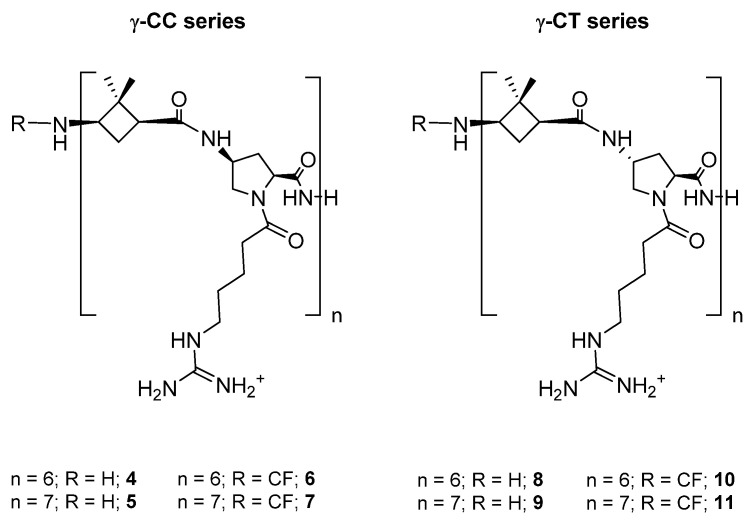
Hybrid peptides of the γ-CC and γ-CT series constituted by repetition of the dipeptide unit γ-CBAA (**1**)/(*cis*- or *trans*)-γ-amino-l-proline, respectively.

The aim of the current work is the validation of [(1*S*,3*R*)-γ-CBAA]-based peptides as vectors in DDS for *Leishmania* chemotherapy, with two major advantages. The first one comprises circumventing the high proteolytic environment associated with this parasite throughout its life cycle; the second is the ability to take advantage of the anionic surface of *Leishmania* to achieve a higher specific recognition by cationic peptides compared to mammalian cells. To this end, two series of diastereomeric peptides of variable length, formed by the alternation of **1** with *N*^α^-substituted *cis*- or *trans*-γ-amino-l-proline, respectively (Chart 1), were synthesized in order to define the length and stereochemistry as the main descriptors for their toxicity and internalization, both on *Leishmania* and HeLa cells, as well as their ability as DDS by conjugation of Dox.

Moreover, the rationale of their cell-transfection ability was examined by studying their structural features through a combination of circular dichroism (CD) spectroscopy and computational calculations.

In all, ((1*S*,3*R*)-γ-CBAA)-based peptides afforded better results than TAT (transactivator of transcription) as vectors for anti-*Leishmania* DDS, opening a new avenue for the implementation of CPP in the chemotherapy against this important protozoan disease.

## 2. Results and Discussion

### 2.1. Design and Synthesis of Peptides and Conjugates

Two series of dodecameric and tetradecameric hybrid oligomers (**4**–**11**) were synthesized. They were formed by the repetition of a dipeptide motif made up by (1S,3R)-γ-CBAA, **1**, [21] linked to a conveniently functionalized cis- or trans-γ-amino-l-proline (γ-CC series and γ-CT series, respectively), (Chart 1). All of them were prepared using standard protocols of solid phase peptide synthesis (SPPS), either in their free *N*-terminus form or functionalized with a carboxyfluorescein (CF) fluorescent group, as detailed at the Section 3 and the Supplementary Materials (SM).

TAT_48–57_ (TAT) [53,54,55] was also synthesized as a reference CPP, either with a free or carboxyfluoresceinated *N*-terminus (see Supplementary Materials).

**Scheme 1:**
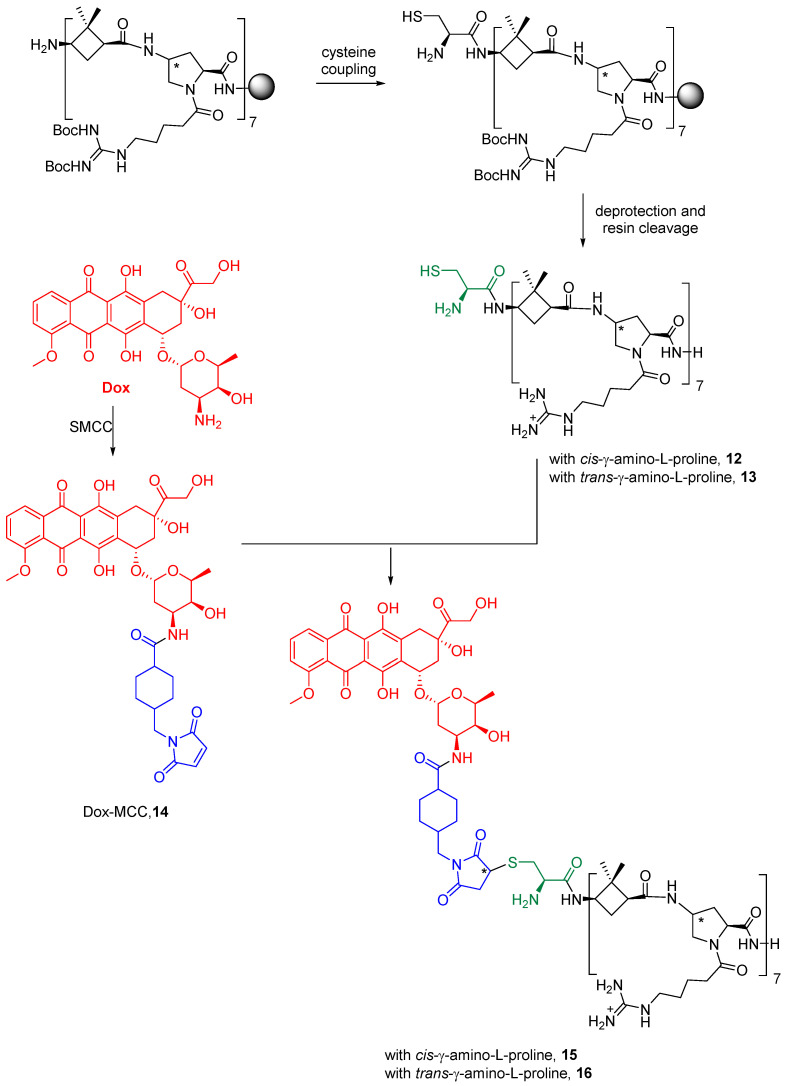
Conjugation of doxorubicin to γ-CC **15** and γ-CT **16** peptides. The Dox moiety is highlighted in red, the cysteine residue in green, and the linker in blue.

Dox was covalently conjugated to γ-CC and γ-CT tetradecameric peptides (Scheme 1), due to their higher internalization rates in *Leishmania* (Section 2.3). For this purpose (Scheme 1), a cysteine was added to the *N*-terminal end of the peptides to obtain **12** and **13**. Then, they were conjugated to doxorubicin, previously functionalized as 4-(*N*-maleimidomethyl)cyclohexane-1-carboxylate (MCC), **14**, (conjugates **15** and **16**). The same conjugation procedure was applied to TAT (see Supplementary Materials for details). To note, a new stereogenic center (marked with an asterisk) was created in the succinimide ring of the linker in the last synthetic step of Dox conjugates, giving two epimers that were used as a mixture. Their influence on the preferred conformations of these conjugates is discussed in Section 2.5 and in the Supplementary Materials.

### 2.2. Cytotoxicity and Cellular Uptake in HeLa Cells

**Figure 2:**
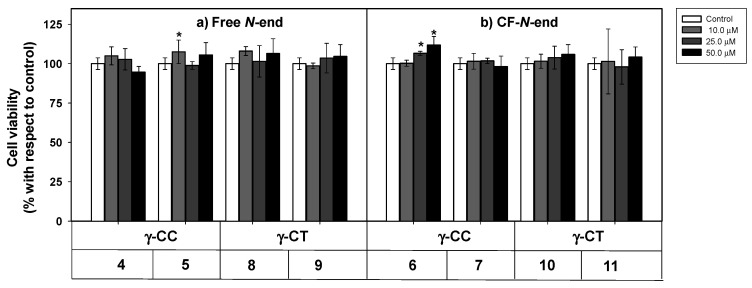
Cytotoxicity of peptides **4**–**11** on HeLa cells. Peptides with (**a**) terminal free amino group; (**b**) terminal amino group conjugated with CF. Cells were incubated with the respective peptide for 24 h and their viability assayed by 3-(4,5-dimethylthiazol-2-yl)-2,5-diphenyltetrazolium bromide (MTT) reduction. Cell viability was expressed as the percentage of MTT reduction with respect to control cells ± SD. Statistical significance with respect to control cells: *p* ≤ 0.05 (*). Samples were made in quadruplicate, and experiments were repeated thrice independently.

As a first step to establish the soundness of γ-CC and γ-CT peptides as therapeutic CPP for *Leishmania*, peptides **4**–**11** were assayed for their toxicity on HeLa cells, used as a model of mammalian cells. The cellular viability after 24 h incubation with the respective peptide was assessed by the reduction of 3-(4,5-dimethylthiazol-2-yl)-2,5-diphenyltetrazolium bromide (MTT) [56] (Figure 2).

Even at the highest concentration assayed (50 µM), viability was over 90%, regardless of the peptide stereochemistry (Figure 2). Peptide toxicity was not dependent either on the number of guanidinium groups in the sequence, or on the carboxyfluoresceination of the terminal amino group. In this sense, these results agree with the low toxicity of polyarginine peptides (R)_n_, up to *n* ≤ 10 [57].

**Figure 3:**
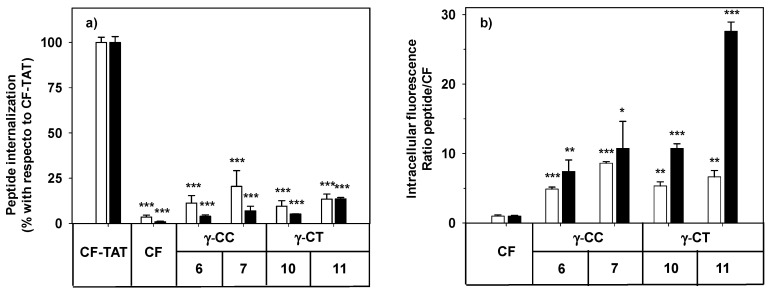
Cellular internalization of carboxyfluoresceinated peptides **6**, **7**, **10**, and **11** normalized (**a**) with respect to CF-TAT (100%), and (**b**) as a fluorescence ratio of the peptides with respect to CF as a control of endocytosis (CF fluorescence = 1 a.u.). HeLa cells were incubated with the respective peptide at 10 (empty column), or 25 µM (black column) for 24 h, and the level of fluorescence associated with the cells was assessed by flow cytometry (λ_EXC_ = 488 nm and λ_EM_ = 530 nm). Results were expressed as mean ± SD. Samples were made in triplicate (*p* ≤ 0.05, (*); *p* ≤ 0.01, (**); *p* ≤ 0.001, (***)). Three independent experiments were carried out.

By flow cytometry, more than 98% of the HeLa cells increased their respective cell-associated fluorescence after incubation with peptides **6**, **7**, **10** and **11** at the range of concentrations tested (Figure 3). The mean of the population represented in Figure 3 refers either to CF-TAT as a standard CPP, or to CF as an endocytosis marker.

The facts that the performance of TAT on HeLa cells does not increase linearly with peptide concentration (10 μM and higher), and that the cell population becomes heterogeneous with respect to peptide uptake [58], could explain the apparently anomalous results obtained when CF-TAT was used as a reference (Figure 3a). In contrast, the cellular fluorescence for both the γ-CT and γ-CC peptide series increased with concentration and with the length of the peptide when using CF as a control of endocytosis (Figure 3b). Interestingly, in all cases, the fluorescence values for the γ-CT peptides were higher than those of the respective γ-CC homologues.

**Figure 4:**
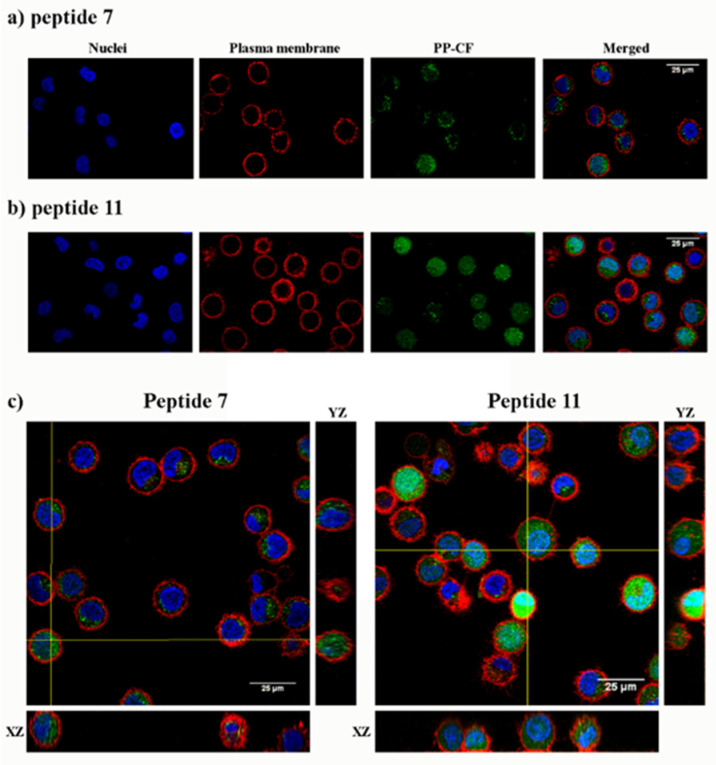
Confocal microscopy of HeLa cells incubated with the carboxyfluoresceinated γ-CC **7** and γ-CT **11** tetradecameric peptides. (**a**) Peptide γ-CC **7**. (**b**) Peptide γ-CT **11**. (**c**) Orthogonal projections highlight the internalization (XZ and YZ) of both peptides. HeLa cells were incubated with the respective CF peptides (2 h, 37 °C; 25 µM), (green fluorescence λ_EXC_ = 488 nm; λ_EM_ = 510 nm), and additionally stained with CellMask, deep red (red fluorescence, λ_EXC_ = 658 nm; λ_EM_ = 690 nm) and Hoechst (blue fluorescence, λ_EXC_ = 405 nm; λ_EM_ = 460 nm) as a plasma membrane and nucleic acids markers, respectively. Magnification bar: 25 µm.

The cell internalization of peptides **7** and **11** in HeLa cells was evidenced by confocal microscopy under identical conditions to flow cytometry. The intracellular fluorescence showed a spotted cytoplasmic pattern after incubation with the peptides at 25 μM peptide (Figure 4). Confocal 3D ruled out their mere association with the plasma membrane. 

### 2.3. Uptake, Microbicidal Activity and Intracellular Location of Peptides on Leishmania Parasites

Once the lack of toxicity of γ-CPP and their low uptake in mammalian cells compared to TAT had been demonstrated, their performance in *Leishmania* was examined as a model of microorganisms with anionic plasma and endowed with a high proteolytic level. To this end, peptides were assayed for viability, uptake and location on the protozoan *Leishmania* parasites.

**Figure 5:**
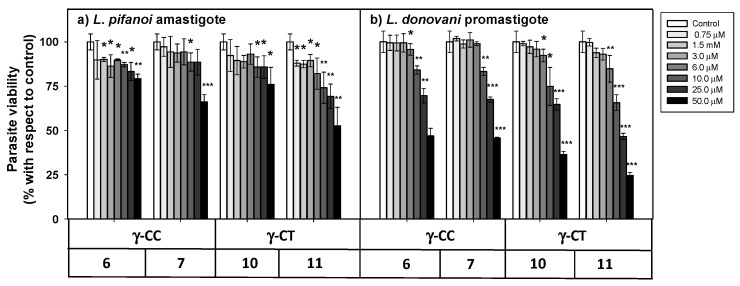
Viability of *Leishmania* sp. parasites after 4 h incubation with peptides **6, 7, 10** and **11**. (**a**) *L. pifanoi* axenic amastigotes. (**b**) *L. donovani* promastigotes. Axenic *Leishmania* parasites (20 × 10^6^ cells/mL) were incubated with the peptides, and MTT reduction measured immediately after 4 h incubation. Viability was represented as the percentage of MTT reduction (mean ± SD). Statistical significance with respect to control parasites: *p* ≤ 0.05, (*); *p* ≤ 0.01, (**); *p* ≤ 0.001, (***). Results are representative of one out of three independent experiments. Samples were made in triplicate.

The cellular viability in the presence of the peptides was assessed by the MTT reduction at both stages of the parasite after 4 h incubation (Figure 5).

The loss of parasite viability due to peptides was concentration-dependent, with promastigotes (Figure 5b) being more susceptible than amastigotes (Figure 5a). The γ-CT series showed higher toxicity than its respective γ-CC counterparts. This trend was especially noticeable for peptide concentrations over 25 μM. Concerning toxicity, the tetradecapeptides (**7** and **11**) were slightly more toxic than the corresponding dodecamers (**6** and **10**), likely due to the increase of their cationic character [59]. Thus, these γ-CPP behave as mild leishmanicidal agents.

**Figure 6:**
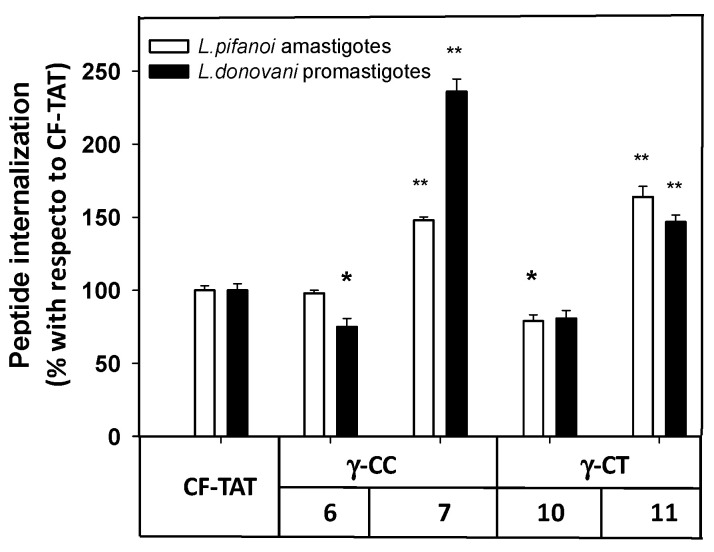
Flow cytometry of carboxyfluoresceinated **6**, **7**, **10** and **11** and TAT_48–57_ peptides associated with *Leishmania pifanoi* amastigotes and *Leishmania donovani* promastigotes. Parasites (20 × 10^6^ cells/mL) were incubated for 4 h with the respective CF-peptides at 10 μM. Afterwards, peptide association to parasites was assessed by flow cytometry (λ_EXC_ = 488 nm, λ_EM_ = 519 nm) and represented as the percentage with respect to TAT-CF ± SD. White bars: *Leishmania pifanoi* axenic amastigotes; black bar: *Leishmania donovani* promastigotes. Statistical significance was referred with respect to the uptake of carboxyfluoresceinated TAT: *p* ≤ 0.05, (*); *p* ≤ 0.01, (**). Results are representative of one out of three independent experiments. Samples were made in triplicate.

Next, the internalization of the carboxyfluoresceinated CPP into promastigotes and amastigotes was measured by flow cytometry, and their values compared to that of CF-TAT (100%). As shown in Figure 6, a similar trend was observed for both parasite systems. The fluorescence values of dodecamers **6** and **10** were relatively similar or up to 30% lower than that of CF-TAT, while parasites incubated with tetradecamers **7** and **11** showed higher fluorescence than CF-TAT.

Under the same conditions, the intensity of the fluorescence was much higher in promastigotes than in amastigotes (data not shown), likely due to their much smaller cellular size; consequently, the promastigotes were chosen for the studies with confocal microscopy.

Thus, these γ-CPP are potential cargo carriers at concentrations ≤ 25 μM; over this value, they behave as leishmanicidal agents on their own. This, together with their nil toxicity and poor uptake in mammalian cells, makes them amenable as DDS systems.

**Figure 7:**
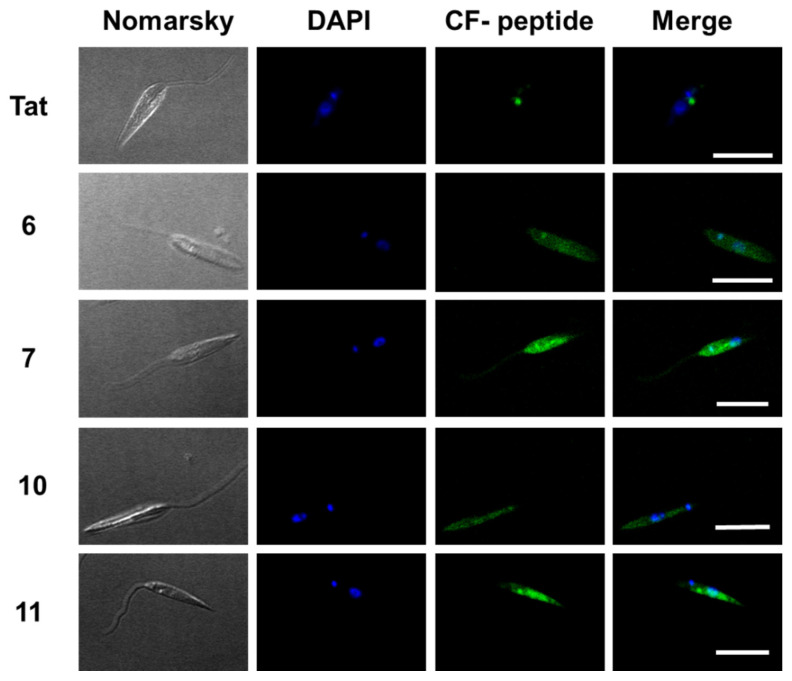
Confocal microscopy of *Leishmania donovani* promastigotes incubated with the selected γ/γ-peptides and TAT. Parasites were incubated with the corresponding peptides (10 μM final concentration, 2 h, 26 °C). Afterwards, they were observed without further fixation. CF-peptides (green fluorescence: λ_EXC_ = 488 nm/λ_EM_ = 519 nm). DAPI (DNA probe, blue fluorescence: λ_EXC_ = 358 nm/λ_EM_ = 461 nm). Magnification bar = 10 μm.

The internalization of these peptides in *Leishmania donovani* promastigotes was assessed by confocal microscopy on those peptides with the best results in flow cytometry. Thus, promastigotes were incubated with peptides **6**, **7**, **10** and **11** as well as TAT (Figure 7) at a final concentration of 10 μM for 2 h at 26 °C. DAPI was used as a nucleic acid dye to stain the nucleus and the kinetoplast (blue fluorescence) (Figure 7).

According to Figure 7, the peptides accumulated inside the promastigote. The pattern was not homogenous. Some dotted areas showed higher fluorescence intensity, with a special relevance near the flagellar pocket, the specialized area of *Leishmania* in charge of all endocytic and exocytic traffic, indicating endocytic CPP uptake. Under these conditions, the nucleus excluded peptides.

In contrast to HeLa uptake, γ-CPP exceeded TAT uptake on *Leishmania*. In other words, an unexpected selectivity towards the parasite was found for these peptides, which supports their potential as selective vectors in DSS for this protozoan in its vertebrate host. A higher selectivity threshold for DSS against the parasite is of special relevance, due to the feasible avoidance of side-effects associated with the current leishmanicidal drugs in *Leishmania* [60], and the probable lowering of the selectivity index for the payload with respect to its administration as free drug.

**Figure 8:**
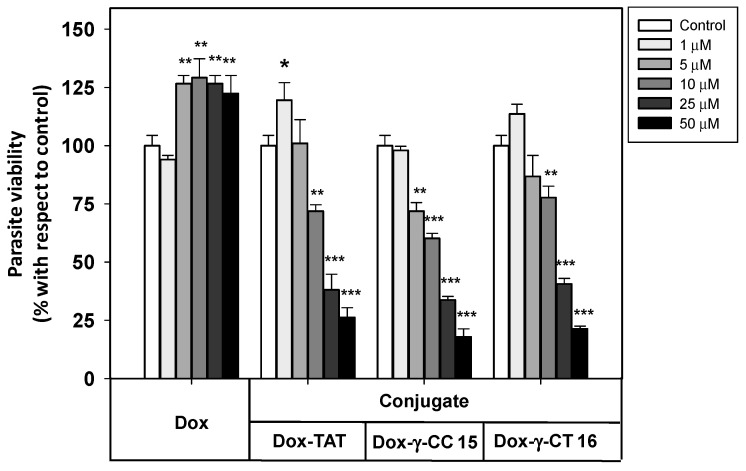
Viability of *Leishmania donovani* promastigotes treated with Dox-conjugates **15** and **16** along with Dox and Dox-TAT. Parasites were incubated with the respective reagents (20 × 10^6^ cells/mL, 4 h, 26 °C), and MTT reduction measured afterwards. Parasite viability was represented as the percentage of MTT reduction ± SD with respect to untreated parasites. Statistical significance with respect to control parasites: *p* ≤ 0.05 (*); *p* ≤ 0.01 (**); *p* ≤ 0.001 (***). Samples were made in triplicate. Results from one out of three independent experiments were represented.

Due to their higher accumulation both in *Leishmania* and HeLa cells, tetradecamers **7** and **11** were selected as vehicles for Dox-conjugation (conjugates **15** and **16**, respectively), in order to be applied as DDS on *Leishmania.* Figure 8 shows the viability of the parasites when treated with these conjugates.

Free doxorubicin did not show any toxicity towards *Leishmania donovani* promastigotes at the full range of concentrations assayed, which avoided the leishmanicidal effect caused by free doxorubicin at longer incubation times (<12 h).

In contrast, the Dox-conjugates **15** and **16** showed an increasing toxicity at concentrations ≥ 1 μM. The peptide γ-CC **15** was shown to be slightly more toxic than γ-CT **16** at 5, 10 and 25 μM. MD simulations could account for such a difference (see below). In addition, Dox-TAT toxicity was like that of **15** and **16**.

**Figure 9:**
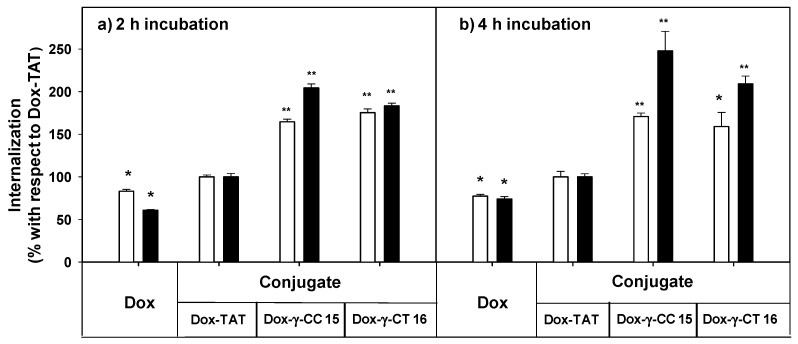
Uptake of free- and peptide-conjugated doxorubicin by *Leishmania donovani* promastigotes at (**a**) 2 h incubation, (**b**) 4 h incubation. Doxorubicin uptake was quantified by flow cytometry using the doxorubicin fluorescence setting (λ_EXC_ = 488 nm; λ_EM_ = 515 nm) after incubation of the parasites (20 × 10^6^ cells/mL, 26 °C in HBSS-Glc) for (**a**) 2 h, and (**b**) 4 h. Data were represented as percentage ± SD with respect to the uptake of the Dox-TAT conjugate. Reagent concentration: 5 μM (white columns); 10 µM (black columns). Samples were made by duplicate (*p* ≤ 0.05, (*); *p* ≤ 0.01, (**)). Dox: doxorubicin; HBSS-Glc: Hanks buffered saline solution and glucose.

The uptake of free doxorubicin and of their peptide conjugates in *L. donovani* promastigotes was quantified by flow cytometry at 5 and 10 μM, measured at 2 and 4 h incubations (Figure 9).

Dox-conjugates **15** and **16** showed better internalization than Dox-TAT, a trend that was observed with parent peptides **7** and **11** (see Figure 6) despite the modifications introduced (doxorubicin conjugation plus the additional cysteine). Longer incubation did not substantially improve the uptake for **15** and **16** at 5 μM, but it did at 10 μM. Under these conditions, the accumulation of **15** was 2.5-fold higher than that of Dox-TAT.

### 2.4. CD Spectroscopy

The CD of γ-CC **5** and γ-CT **9** conjugates showed respective monosignated spectra with λ_max_ at 215 and 212 nm (see Figure S1 in Supplementary Materials); nevertheless, an accurate insight into their folding from these data was insufficient, even when some defined conformational bias for oligomers consisting of γ-amino acids was published [61]. To make these aspects clear, molecular modeling studies were undertaken.

### 2.5. Molecular Modeling

The hydrophobicity of amphipathic CPP [62,63] is crucial for their high affinity binding to lipid membranes, including those from internalized vesicles [64,65]. In addition, the creation of high-density positive areas in amphipatic cationic peptides favors their specific binding into anionic biopolymers, such as the cell membranes of pathogens [66].

Therefore, molecular dynamics (MD) simulations were carried out on γ-CC **5** and γ-CT **9** peptides under an explicit solvent scenario. The goals were to shed light on the folding of peptides in aqueous solution and to determine how the arrangement of positive charges could be involved in cellular binding. In addition, the role of their respective cargo molecules (doxorubicin and carboxyfluorescein) in the conformation of the conjugates was also examined. For doxorubicin conjugates, both (*R*) and (*S*) epimers were considered (see Supplementary Materials for details).

**Figure 10:**
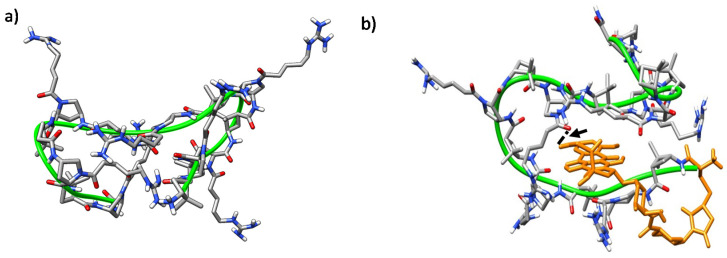
Representative MD conformations for (**a**) peptide γ-CC **5** and (**b**) conjugate (*R*)-Dox-γ-CC, (*R*)-**15**. The peptide scaffold is highlighted by the green ribbon; Dox is represented in orange; the arrow points out long-range hydrogen-bonding between the primary hydroxyl group of the cyclohexane side-chain in Dox moiety and the *N*^α^-side chain C*O* in proline residue *i* = 8.

The trajectories of both CC and CT diastereomeric peptides converged into defined folded states after ~40 ns. In all of them, a hydrophobic core was formed and the polar guanidinium groups of the positively charged residues faced the solvent. A double hairpin motif was predominant for γ-CC **5** (Figure 10a), promoted mostly by diverse inter-residue hydrogen-bonds (Figure S7a). These hairpins, and also β-sheet-like structures, have been observed for similar peptides based on *cis*-γ-amino-l-proline [67]. For the conjugate (*R*)-Dox-γ-CC, (*R*)-**15**, a stable conformation occurred after ~75 ns, with the Dox moiety being partially buried in the main peptide scaffold (Figure 10b). This perturbation of the original double hairpin by the presence of Dox is the consequence of long-range hydrogen-bonding between the primary hydroxyl group of the cyclohexane side-chain in the Dox moiety and *N*^α^-side-chain C*O* in proline residue *i* = 8 (Figure S7b).

A related conformation was the most stable for epimer (*S*)-Dox-γ-CC, (*S*)-**15**; the Dox moiety was folded towards the peptide backbone cavity with the establishment of a long-range hydrogen bond between the hydroxyl group of the amino sugar ring of the Dox-moiety and proline residue *i* = 12 (Figures S6 and S7c). The hairpin-like conformation was also obtained, along with a laminar one, when the conjugate CF-γ-CC **7** was considered (Figures S8 and S9).

**Figure 11:**
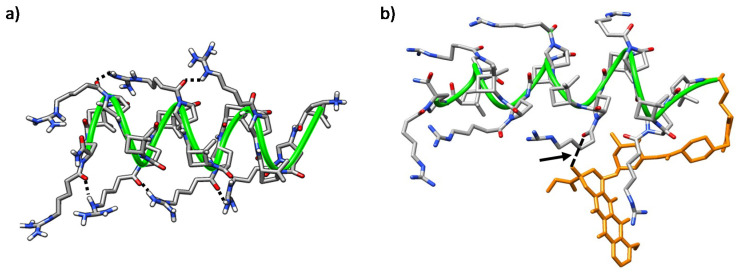
Representative MD conformations for (**a**) peptide γ-CT **9**, inter-residue interactions are marked; and (**b**) conjugate (*R*)-Dox-γ-CT, (*R*)-**16**. The peptide scaffold is highlighted by the green ribbon; Dox is represented in orange; the arrow points out long-range hydrogen-bonding between the tertiary hydroxyl group of the cyclohexane ring in the Dox-moiety and the *N*^α^-side chain C*O* in proline residue *i* = 6.

On the other hand, peptide γ-CT **9** adopted a stable right-handed helix structure that was massively occupied (77%) along the trajectory (Figure 11a herein and Figure S10). This folding was driven by intra-residue hydrogen bonds formed by the N*H* and the carbonyl group in the γ-CBAA units, and inter-residue ones formed between the protons of the guanidinium group in an *i* residue and the proline *N*^α^-side-chain C=*O* at I + 4 (Figure 11a).

Helical conformations were also predominant in both (*R*) and (*S*) isomers of conjugate Dox-γ-CT **16**; in both cases, the Dox moiety faced the peptide backbone, becoming partially hidden. Nevertheless, this interaction is dependent on the configuration at the epimeric center of the linker. In the (*R*) epimer, a relevant long-range hydrogen bond between the hydroxyl group of the Dox cyclohexane moiety with the *N*^α^-side-chain C=*O* in proline residue *i* = 6 was established (Figure 11b and Figure S11b). In contrast, this hydrogen bond was not relevant for the (*S*) epimer (Figure S11a,c), and doxorubicin was buried in the core of the peptide, probably to minimize its hydrophobic interactions with the aqueous medium. This feature could account for the slight difference of toxicity observed for both conjugates Dox-γ-CC **15** and Dox-γ-CT **16**, as noted in Section 2.3.

Moreover, the stability of the helical structure of CF-γ-CT **11** was maintained along 450 ns in the simulation, despite its CF terminal moiety, located at the periphery of the peptide backbone (Figure S12).

Therefore, MD highlights the critical role of the *cis*/*trans* stereochemistry of the γ-amino-l-proline monomer for the folding of γ-CC and γ-CT peptide series. Although a specific conformation is not unequivocally associated with a better uptake, good cellular uptake has been reported for helical, hairpin and sheet-like peptides [34,68]. In addition, it is well documented that the delocalized charge of the guanidinium group endows a high membrane-translocation efficiency upon a variety of arginine-rich CPP [68,69,70]. In our case, the conjugation of doxorubicin to the *N*-terminus of the peptide induced the hydrophobic collapse of the skeleton around the drug, which become buried, regardless of the diastereomeric series or the configuration of epimeric center. As this folding exposes the guanidinium group of the side-chains to the external aqueous medium, better membrane translocation is assumed.

## 3. Materials and Methods

### 3.1. Synthesis of the γ-CC Peptides (***4***–***7***), γ-CT Peptides (***8***–***11***) and TAT_48–57_ Peptides

Resin H-rink amide ChemMatrix^®^ (Sigma Aldrich, Barcelona, Spain) with 0.47 mmol/g functionalization was used, previously conditioned by several washes with DMF (dimethylformamide) and DCM (dichloromethane) (Sigma Aldrich, Barcelona, Spain). The synthesis of both hybrid peptide series and TAT_58–57_ was performed by using the Fmoc/Alloc strategy, although monomers for TAT_48–57_ contained other protecting groups. See the Supplementary Materials for more details.

### 3.2. Synthesis of Cysteine Elongated Peptides (***12***) and (***13***)

The peptides were prepared following the protocol outlined for peptides **5** and **9**. The peptides were elongated with a Cys(Trt) residue as detailed in the Supplementary Materials.

### 3.3. Synthesis of Doxorubicin-MCC (***14***)

Doxorubicin·HCl salt (3 mg, 5.17 µmol), succinimidyl 4-(*N*-maleimidomethyl)cyclohexane-1-carboxylate (SMCC) (2.1 mg, 6.60 µmol) and diisopropylethylamine (5 µL, 7.75 µmol) were added to 450 µL DMF in a 2 mL round-bottomed flask. The reaction was allowed to run in the dark at room temperature. The reaction was followed by RP-HPLC-MS. After 1.5 h, 450 µL of PBS were added and the pH was lowered to 6–7 with 1 M HCl. *m*/*z* (ESI): Ms Calcd for C_39_H_42_N_2_O_14_ [M+H]^+^: 763.4; Experimental: 763.1.

### 3.4. Synthesis of Conjugates Dox-γ-CC (***15***) and Dox-γ-CT (***16***)

1.2 Equivalents of peptide **12** or **13**, respectively, were added to the reaction mixture containing **14**, and the reaction was stirred at room temperature until the reactants were consumed (determined by RP-HPLC-MS). After the reaction, the crude product was immediately purified by RP-HPLC.

### 3.5. Peptide and Conjugate Purification

Semiprep-RP-HPLC-MS was used to purify the peptides, formed by a binary gradient Waters 2545, a Waters Alliance 2767 sample manager module and an automatic fraction collector coupled to a dual UV-vis λ Absorbance Detector 2487 and a mass spectrometer Micromass ZQ. An XBridge^®^ Prep BEH C18 (19 × 100 mm, 5 μm) column (Waters, Cerdanyola del Vallès, Spain) was used. The eluents were A: ACN/HCOOH (99.93:0.07, *v*/*v*) and B: H_2_O/HCOOH (99.9:0.10, *v*/*v*). The column was preconditioned by washing with buffer A for 10 min. Gradient conditions are described below.

### 3.6. Peptide Characterization

For the analysis and determination of the retention times (t_R_) of each peptide, a linear gradient of 0% to 50% of B was used for 15 min in the column XBridge™ BEH 130 C18 (4.6 × 100 mm, 3.5 μM) (Waters, Cerdanyola del Vallès, Spain) with a 1 mL/min flow. For peptide **7**, a linear gradient of 0% to 100% of A for 15 min was used. The elution system used was A: H_2_O/TFA (99.90:0.10, *v*/*v*) and B: ACN/HCOOH (99.90:0.10, *v*/*v*). The purity of the compounds was determined at λ = 220 nm

#### 3.6.1. Peptide γ-CC (**4**)

Purification: RP-HPLC-MS gradient: Increase from 7% to 10% of ACN/HCOOH in 7 min; from 10% to 100% of ACN/HCOOH in 0.5 min (maintained for 2 min). Then, initial conditions were reestablished after 0.5 min. Characterization: RP-HPLC Purity >95% and retention time: 6.0 min. *m*/*z* (ESI): Ms Calcd for C_108_H_188_N_37_O_18_ [(M+5H)/5]^+^: 458.5; Experimental: 458.6. Ms Calcd for C_108_H_189_N_37_O_18_ [(M+6H)/6]^+^: 382.3; Experimental: 382.3; Ms Calcd for C_108_H_190_N_37_O_18_ [(M+7H)/7]^+^: 327.8; Experimental: 327.8.

#### 3.6.2. Peptide γ-CC (**5**)

Purification: RP-HPLC-MS gradient: Increased from 5 to 12% ACN/HCOOH in 7 min, increased from 12% to 100% in 0.5 min, then maintained for 2 min. Then the initial conditions were reestablished. Characterization: RP-HPLC. Purity >97% and retention time: 6.2 min. *m*/*z* (ESI): Ms Calcd for C_126_H_219_N_43_O_21_ [(M+6H)/6]^+^: 445.4; Experimental: 445.4. Ms Calcd for C_126_H_220_N_43_O_21_ [(M+7H)/7]^+^: 381.9; Experimental: 381.9. Ms Calcd for C_126_H_221_N_43_O_21_ [(M+8H)/8]^+^: 334.3; Experimental: 334.3.

#### 3.6.3. Peptide CF-γ-CC (**6**)

Purification: RP-HPLC-MS gradient: from 10% to 20% ACN/HCOOH in 7 min; and to 100% of ACN/HCOOH in 0.5 min, kept for 2 min, and returned to starting conditions after 0.5 min. Characterization: RP-HPLC. Purity >95% and retention time: 7.7 min. *m*/*z* (ESI): Ms Calcd for C_129_H_198_N_37_O_24_ [(M+5H)/5]^+^: 530.2; Experimental: 530.3. Ms Calcd for C_129_H_199_N_37_O_24_ [(M+6H)/6]^+^: 442.0; Experimental: 442.0; Ms Calcd for C_129_H_200_N_37_O_24_ [(M+7H)/7]^+^: 379.0; Experimental: 379.0.

#### 3.6.4. Peptide CF-γ-CC (**7**)

Purification: RP-HPLC-MS gradient: Increase from 12 to 20% ACN/HCOOH in 7 min, and to 100% after an additional 0.5 min, then kept for 2 min. Afterwards, starting conditions were reestablished. Characterization: RP-HPLC. Purity >98% and retention time: 5.1 min. *m*/*z* (ESI): Ms Calcd for C_147_H_230_N_43_O_27_ [(M+6H)/6]^+^: 505.1; Experimental: 505.1. Ms Calcd for C_147_H_231_N_43_O_27_ [(M+7H)/7]^+^: 433.1; Experimental: 433.1. Ms Calcd for C_147_H_232_N_43_O_27_ [(M+8H)/8]^+^: 379.0; Experimental: 379.0.

#### 3.6.5. Peptide γ-CT (**8**)

Purification: RP-HPLC-MS gradient: Increase from 7 to 10% ACN/HCOOH in 7 min, and to 100% in 0.5 min, then kept for 2 min, and initial conditions restored. Characterization: RP-HPLC. Purity >96% and retention time: 5.8 min. *m*/*z* (ESI): Ms Calcd for C_108_H_188_N_37_O_18_ [(M+5H)/5]^+^: 458.5; Experimental: 458.5. Ms Calcd for C_108_H_189_N_37_O_18_ [(M+6H)/6]^+^: 382.3; Experimental: 382.3. Ms Calcd for C_108_H_190_N_37_O_18_ [(M+7H)/7]^+^: 327.8; Experimental: 327.8.

#### 3.6.6. Peptide γ-CT (**9**)

Purification: RP-HPLC-MS gradient: Increase from 5 to 12% ACN/HCOOH in 7 min, and to 100% in 0.5 min, kept for 2 min, and then, initial conditions were reestablished. Characterization: RP-HPLC. Purity >96% and retention time: 5.9 min. *m*/*z* (ESI): Ms Calcd for C_126_H_219_N_43_O_21_ [(M+6H)/6]^+^: 445.4; Experimental: 445.3. Ms Calcd for C_126_H_220_N_43_O_21_ [(M+7H)/7]^+^: 381.9; Experimental: 381.9. Ms Calcd for C_126_H_221_N_43_O_21_ [(M+8H)/8]^+^: 334.3; Experimental: 334.2.

#### 3.6.7. Peptide CF-γ-CT (**10**)

Purification: RP-HPLC-MS gradient: Increase from 10 to 20% ACN/HCOOH in 7 min, and to 100% in 0.5 min, then kept for 2 min. Then, the initial conditions are reestablished. Characterization: RP-HPLC. Purity >97% and retention time: 8.0 min. *m*/*z* (ESI): Ms Calcd for C_129_H_196_N_37_O_24_ [(M+3H)/3]^+^: 883.0; Experimental: 883.4. Ms Calcd for C_129_H_198_N_37_O_24_ [(M+5H)/5]^+^: 530.2; Experimental: 530.3. Ms Calcd for C_129_H_199_N_37_O_24_ [(M+6H)/6]^+^: 442.0; Experimental: 442.1. Ms Calcd for C_129_H_200_N_37_O_24_ [(M+7H)/7]^+^: 379.0; Experimental: 379.0.

#### 3.6.8. Peptide CF-γ-CT (**11**)

Purification: RP-HPLC-MS gradient: Increased from 12 to 20% ACN/HCOOH in 7 min, and to 100% in 0.5 min, then kept for 2 min and initial conditions were reestablished. Characterization: RP-HPLC. Purity >99% and retention time: 7.9 min. *m*/*z* (ESI): MS Calcd for C_147_H_230_N_43_O_27_ [(M+6H)/6]^+^: 505.1; Experimental: 505.1. Ms Calcd for C_147_H_231_N_43_O_27_ [(M+7H)/7]^+^: 433.1; Experimental: 433.2. Ms Calcd for C_147_H_232_N_43_O_27_ [(M+8H)/8]^+^: 379.0; Experimental: 379.0.

#### 3.6.9. TAT_48–57_

Purification: RP-HPLC-MS gradient: 0% ACN/HCOOH for 4 min, from 0% to 4% ACN/HCOOH in 3 min; and to 100% ACN/HCOOH in 0.5 min, then kept for 2 min. Then, the initial conditions were reestablished. Characterization: RP-HPLC. Purity >97% and retention time: 4.7 min. *m*/*z* (ESI): Ms Calcd for C_55_H_114_N_32_O_11_ [(M+4H)/4]^+^: 349.7; Experimental: 349.9. Ms Calcd for C_55_H_115_N_32_O_11_ [(M+5H)/5]^+^: 279.9; Experimental: 280.0. Ms Calcd for C_55_H_116_N_32_O_11_ [(M+6H)/6]^+^: 233.4; Experimental: 233.5.

#### 3.6.10. TAT_48–57_-CF

Purification: RP-HPLC-MS gradient using from 0% to 7% ACN/HCOOH in 7 min; from 7% to 100% ACN/HCOOH in additional 0.5 min, then kept for 2 min. Then the initial conditions were reestablished. Characterization: RP-HPLC. Purity >99% and retention time: 7.0 min. *m*/*z* (ESI): Ms Calcd for C_76_H_124_N_32_O_17_ [(M+4H)/4]^+^: 439.2; Experimental: 439.4. Ms Calcd for C_76_H_125_N_32_O_17_ [(M+5H)/5]^+^: 351.6; Experimental: 351.8. Ms Calcd for C_76_H_126_N_32_O_17_ [(M+6H)/6]^+^: 293.2; Experimental: 293.2.

#### 3.6.11. Peptide (**12**)

Purification: RP-HPLC-MS gradient: Increase from 5 to 12% ACN/HCOOH in 7 min, and from 12 to 100% in 0.5 min, then kept for 2 min, and return to initial conditions. Characterization: RP-HPLC. Purity >85% and retention time: 6.7 min. *m*/*z* (ESI): Ms Calcd for C_131_H_224_N_44_O_23_S [(M+4H)/4]^+^: 703.7; Experimental: 703.7. Ms Calcd for C_131_H_225_N_44_O_23_S [(M+5H)/5]^+^: 563.2; Experimental: 563.2. Ms Calcd for C_131_H_226_N_44_O_23_S [(M+6H)/6]^+^: 469.4; Experimental: 469.4. Ms Calcd for C_131_H_227_N_44_O_23_S [(M+7H)/7]^+^: 402.5; Experimental: 402.5.

#### 3.6.12. Peptide (**13**)

Purification: RP-HPLC-MS gradient: from 5 to 12% ACN/HCOOH in 7 min, from 12 to 100% in additional 0.5 min, then kept for 2 min, and initial conditions were reestablished. Characterization: RP-HPLC. Purity >96% and retention time: 6.2 min. *m*/*z* (ESI): Ms Calcd for C_131_H_224_N_44_O_23_S [(M+4H)/4]^+^: 703.7; Experimental: 703.6. Ms Calcd for C_131_H_225_N_44_O_23_S [(M+5H)/5]^+^: 563.2; Experimental: 563.2. Ms Calcd for C_131_H_226_N_44_O_23_S [(M+6H)/6]^+^: 469.4; Experimental: 469.4. Ms Calcd for C_131_H_227_N_44_O_23_S [(M+7H)/7]^+^: 402.5; Experimental: 402.6

#### 3.6.13. TAT_48–57_-Cys

Purification: RP-HPLC-MS gradient: 0% ACN/HCOOH for 4 min, from 0 to 4% in 3 min, and at 100% in additional 0.5 min, then kept for 2 min. Then, initial conditions were reestablished. Characterization: RP-HPLC. Purity >78% and retention time: 4.8 min. *m*/*z* (ESI): MS Calcd for C_60_H_119_N_33_O_13_S [(M+2H)/2]^+^: 771.3; Experimental: 771.2. Ms Calcd for C_60_H_120_N_33_O_13_S [(M+3H)/3 ]^+^: 514.4; Experimental: 514.4. Ms Calcd for C_60_H_121_N_33_O_13_S [(M+4H)/4]^+^: 386.1; Experimental: 386.1. Ms Calcd for C_60_H_122_N_33_O_13_S [(M+5H)/5]^+^: 309.1; Experimental: 309.1.

#### 3.6.14. Conjugate Dox- γ-CC (**15**)

Peptide **12** was coupled to Dox-MCC **14**. The purification was performed using RP-HPLC with a gradient: from 0% to 50% ACN/HCOOH in 11 min, and to 100% of ACN/HCOOH in 0.5 min, kept for 2 min; finally, initial conditions were recovered after 0.5 min. The purified peptide was characterized by RP-HPLC and RP-HPLC-MS. Purity >95% pure and retention time: 8.9 min. *m*/*z* (ESI): Ms Calcd for C_170_H_266_N_46_O_37_S [(M+4H)/4]^+^: 894.0; Experimental: 894.1. Ms Calcd for C_170_H_267_N_46_O_37_S [(M+5H)/5]^+^: 715.7, Experimental: 715.7; Ms Calcd for C_170_H_268_N_46_O_37_S [(M+6H)/6]^+^: 596.8, Experimental: 596.8; Ms Calcd for C_170_H_269_N_46_O_37_S [(M+7H)/7]^+^: 511.5, Experimental: 511.5.

#### 3.6.15. Conjugate Dox- γ-CT (**16**)

Peptide **13** was coupled to Dox-MCC **14**. The purification was performed using RP-HPLC with a gradient from 0% to 50% ACN/HCOOH in 11 min, and to 100% ACN/HCOOH in 0.5 min, kept for 2 min; finally, initial conditions were recovered after 0.5 mini. The purified peptide was characterized by RP-HPLC and RP-HPLC-MS. Purity > 95% pure and retention time: 9.0 min. *m/z* (ESI): Ms Calcd for C_170_H_267_N_46_O_37_S [(M+5H)/5]^+^: 715.7, Experimental: 715.7; Ms Calcd for C_170_H_268_N_46_O_37_S [(M+6H)/6]^+^: 596.8, Experimental: 596.7; Ms Calcd for C_170_H_269_N_46_O_37_S [(M+7H)/7]^+^: 511.5, Experimental: 511.5.

#### 3.6.16. Dox-TAT_48–57_

Cysteine-elongated TAT_48–57_ was coupled to Dox-MCC **14**. The purification of the peptide was performed by RP-HPLC with a gradient from 0% to 50% ACN/HCOOH in 11 min, and to 100% in additional 0.5 min, then kept for 2 min); finally, initial conditions reached after 0.5 min. The purified peptide was characterized by RP-HPLC and RP-HPLC-MS. Purity >95% pure and retention time: 8.8 min. *m*/*z* (ESI): Ms Calcd for C_99_H_162_N_35_O_27_S [(M+3H)/3]^+^: 768.7, Experimental: 764.7; Ms Calcd for C_99_H_163_N_35_O_27_S [(M+4H)/4]^+^: 576.8, Experimental: 576.6; Ms Calcd for C_99_H_163_N_35_O_27_S [(M+5H)/5]^+^: 461.6, Experimental: 461.6.

### 3.7. Cellular Viability, Internalization, and Localization Experiments with HeLa Cells

The HeLa cell line, derived from a human cervical cancer, was used to perform the biological assays. CPP dissolved in nonsupplemented Minimum Essential Medium (MEM) at their final concentration were sterilized by filtration through a 0.2 µm polycarbonate filter (Whatman^®^ Puradisc, Sigma Aldrich, Barcelona, Spain) filters.

#### 3.7.1. HeLa Cells Culture

Cells were cultured in 25 cm^2^ flasks in MEM (Gibco, Thermo Fisher Scientific, Cornellà de Llobregat, Spain) culture media supplemented with 10% of heat inactivated fetal bovine serum (FBS, Gibco) plus 2 mM l-glutamine (Biowest, Labclinics, Barcelona, Spain) at 37 °C, saturated humidity and 5% CO_2_ (standard conditions).

#### 3.7.2. Cellular Viability

The MTT assay (Sigma Aldrich, Barcelona, Spain) was used to analyze the cellular viability. This method is based on the ability of living cells to reduce the MTT to formazan salts, as a measure of their viability. HeLa cells were seeded into 24-microwell plates at 6 × 10^4^ cells/mL (30,000 cells/well). After 24 h of incubation, the medium was replaced with the respective peptide concentration and incubated for 24 h. Then, the cells were washed three times with Hanks buffered saline solution (HBSS) (Biowest, Labclinics, Barcelona, Spain), and 500 μL of 0.1 mg/mL MTT in HBSS was added to the cell suspension and incubated for 3 h at 37 °C in darkness. The cell layer was dried in darkness, and the resulting formazan solubilized in pure DMSO. The absorbance was measured at 540 nm in an X3 Multilabel Plate Reader coupled to Perkin Elmer 2030 Manager control software. At least three independent experiments with four different replicates of each peptide and concentration were performed. Controls, nontreated cells or cells incubated with CF were included at identical concentrations. The absorbance values of nontreated cells were taken as 100% cellular viability.

#### 3.7.3. Peptide Internalization

For flow cytometry experiments, HeLa cells were seeded into 35 mm culture dishes (2 × 10^5^ cells/dish). After 24 h incubation under standard conditions, culture medium was removed, and cells were incubated for 2 h with the corresponding peptides at 10 and 25 μM. Next, the culture medium was removed, the cells were washed twice with HBSS and trypsinized with 0.5 mL of 0.25% trypsin-EDTA (Gibco, Thermo Fisher Scientific, Cornellà de Llobregat, Spain). After 5 min incubation at 37 °C, 2 mL of MEM + 10% FCS was added to the cells to stop trypsinization and the mixture was centrifuged (5 min, 300× *g*). Cells were then additionally washed with 2 mL of HBSS under the same conditions. Finally, the cell pellet was resuspended in 200 μL of PBS at pH = 6 to detach any peptide adhering to the plasma membrane. To exclude dead cells from gating, 5 µg/mL propidium iodide (PI, Sigma-Aldrich, Barcelona, Spain) was added to the cells immediately before the flow cytometric analysis, carried out in a BD FACSCanto cytometer (Bio-Rad, Alcobendas, Spain) coupled to FACSDiva v7.0 software using 488 nm and 635 nm lasers to excite the peptides and PI, respectively.

A total of 10,000 single cells were analyzed per sample, and at least three independent experiments were performed with each peptide and concentration. Untreated cells (autofluorescence control) and cell cultures incubated with TAT as positive reference CPP, and CF as negative reference, were included in every experiment. The fluorescence intensity of cells treated with CF was taken as one arbitrary unit for experiment normalization. For confocal microscopy, HeLa cells were seeded into glass bottom culture dishes (MatTek, Bratislava, Slovakia) at a density of 2 × 10^5^ cell/dish. After 24 h of incubation, the culture medium was removed, and the cells were incubated for an extra 2 h in the presence of peptides at 25 µM. Then, cells were rinsed three times with PBS, and nuclei and plasma membrane were counterstained with 1 µl/mL of Hoechst 33342 (10 mg/mL, Thermo Fisher, Scientific, Cornellà de Llobregat, Spain) and 1 µl/mL CellMask™ deep red plasma membrane stain (5 mg/mL, Thermo Fisher Scientific, Cornellà de Llobregat, Spain), respectively. Finally, cells were washed with PBS prior to be resuspended in PBS pH = 6.0. The experiments were performed using an Olympus Fluoview FV1000 confocal laser scanning microscope (Olympus Iberia, Hospitalet de Llobregat, Spain) equipped with Olympus Fluoview as control software. The excitation wavelengths used were 405, 488 and 658 nm to visualize the nuclei, the peptides and the plasma membrane, respectively; the wavelength of emission was 460, 510 and 690 nm, respectively. A 3D reconstruction was generated to obtain orthogonal projections using the ImageJ/fiji software.

### 3.8. Cellular Viability, Internalization and Localization Experiments with Leishmania Parasites

The experiments were carried out with *Leishmania donovani* promastigotes (MHOM/SI/00/1S-2D strain) and *Leishmania pifanoi* amastigotes (MHOM/VE/60/Ltrod strain), respectively. Promastigotes were grown at 26 °C in RPMI medium supplemented with 10% of FCS, 2 mM l-glutamine, 20 U/mL unicillin 20 plus 48 µg/mL gentamicin.

#### 3.8.1. Cellular Viability

The viability assays were carried out using the reduction of MTT as described. The parasites were aliquoted into 96-microwell plates at a final concentration of 20 × 10^6^ cells/mL in HBSS supplemented with 10 mM D-glucose. The peptides were incubated for 4 h at the corresponding peptide concentration at 26 °C or 32 °C for promastigotes and axenic amastigotes, respectively. Afterwards, 0.5 mg/mL MTT in HBSS + 10 mM D-glucose was added and cells were incubated for two additional hours. The resulting formazan was solubilized with DMSO (1% final concentration) and read at 595 nm in a Bio-Rad 640 microplate reader.

#### 3.8.2. Peptide Uptake for *Leishmania donovani* Promastigotes

Parasites were resuspended in HBSS + Glc and dispensed in 24-microwell plates (2 mL/well) at a final concentration of 20 × 10^6^ cells/mL. After incubation with the peptides for 4 h at 26 °C, parasites were washed twice with 2 mL of HBSS + Glc plus 1% fatty-acid free bovine seroalbumin in order to remove the noninternalized peptides, and resuspended in the same medium at 1 × 10^6^ cells/mL. Propidium iodide (PI) at a final concentration of 5 µg/mL was added immediately to the flow cytometric analysis to gate the viable cells exclusively. Flow cytometry was carried out in a FC500 flow cytometer, using λ_EXC_ = 488 nm and λ_EM_ = 525 nm for fluoresceinated peptides and λ_EXC_ = 488 nm and λ_EM_ = 515 nm for free- and conjugated doxorubicin.

#### 3.8.3. Intracellular Localization of the Internalized Peptides in *Leishmania donovani* Promastigotes

Parasites (2 × 10^6^ cells/mL in HBSS + Glc, 200 μL) were incubated with the corresponding peptides (2 h, 26 °C). Afterwards, they were collected from the well and washed twice with 2 mL of HBSS + Glc plus 1% bovine seroalbumin fatty-acid free. Prior to carrying out confocal microscopy, parasites were incubated with 10 µL/mL of DAPI (4′,6-diamidine-2′-phenylindole dihydrochloride) for 10 min at 26 °C and washed twice with 2 mL of HBSS + Glc. Living parasites were observed using a Leica SP2 confocal microscope (Leica Microsistemas, S.A., Barcelona, Spain) at λ_EXC_ = 358 nm/λ_EM_ = 461nm.

### 3.9. Circular Dichroism (CD) Spectroscopy Details

CD spectra were recorded on a JASCO-715 spectropolarimeter (Jasco, Madrid, Spain). For all CD analyses, 50 μM solutions of the peptides in PBS were prepared and the CD spectra were measured after 24 h equilibration at room temperature. A quartz cuvette of 0.1 mm optical path length was used. Spectra were recorded from 195 to 260 nm with 1 nm spectral bandwidth, at room temperature, with a time constant of 4 s, a step resolution of 1 nm and 5 repetitions per experiment. Before each measurement, a blank measurement with pure PBS was performed. CD data are given as mean residual molar ellipticities in deg cm^2^ dmol^-1^.

### 3.10. Computational Details

The geometries of the unnatural amino acids were optimized with Gaussian09 revision D01 [71] at the DFT level of theory using the B3LYP functional combined with the 6-31G(d,p) basis set. Frequency calculations were carried out for all the structures in order to characterize them as minima in the potential energy surface (PES). Atomic charges were computed with the restrained electrostatic potential (RESP) protocol [72]. The atom types and force field parameters were assigned through antechamber and parmchk2 included in AmberTools18 [73].

Semi-empirical calculations at PM6 theory level, using G09, were carried out on the peptide structures before running MD minimizations in order to obtain an initial structure avoiding steric clashes.

Molecular Dynamics (MD) simulations were set up solvating the peptide with a cubic box of TIP3P water molecules and neutralizing the total charge with chloride ions (ions94.lib). The AMBER99SB force field [74] was used for the standard residues, while the GAFF force field [75] was adopted for the remaining atoms. MD simulations were carried under periodic boundary conditions with the OpenMM [76] engine using the OMM Protocol [77]. The trajectories convergence was analyzed by RMSD, RMSF, Counting Clustering Method and PCA analysis, considering the full exploration of the conformational space as criterion of convergence (see the Supplementary Materials) [78]. In particular, for the γ-CC (**5**) and γ-CT (**9**) peptides the simulations were carried out along 200 ns. From a structural point of view, the trajectories were analyzed using CPPTraj implemented in AmberTools18.

## 4. Conclusions

Two diastereomeric series of new dodecameric and tetradecameric hybridd γ/γ-peptides, γ-CC and γ-CT, were synthesized and evaluated as CPP, focusing on their toxicity and cell penetrating activity. These series were formed by repetition of a dipeptide unit constituted by a chiral cyclobutane amino acid and either a *cis*- or *trans*-γ-amino-l-proline, respectively. In preliminary studies, they were shown to be innocuous to HeLa cells and to effect moderate cellular uptake. In contrast, the toxicity of both peptide series both on *Leishmania pifanoi* amastigotes and *Leishmania donovani* promastigotes was concentration dependent. Only full viability was preserved under 10 μM. Therefore, these peptides behave as mild leishmanicidal agents at higher concentrations. There is a thin red line separating the CPP from membrane-active antimicrobial peptides. Only their affinity towards the targeted membrane separates both concepts: either they form a stable disruption of the membrane, with irreversible and lethal outcome, or they provoke a mild and reversible distortion to ensure the peptide translocation. In other words, the polarization towards one of these two stages is based on quantitative factors; as such, a continuous spectrum of intermediate stages must be expected. Among these factors, the nature of the peptide and the phospholipid composition of the membrane are crucial to tip the balance towards one phenotype or the other. These subtleties are further highlighted with cationic cell penetrating peptides acting on prokaryotes or lower eukaryotes with a strongly anionic membrane, as happens with *Leishmania*, and especially with the promastigote. This presents a strong anionic glycocalyx and a high percentage of anionic phospholipids in its plasma membrane, which accounts for a higher γ/γ-peptide internalization than in the amastigote. The relevance of this interaction is endorsed by the higher activity of the tetradecamer over the dodecamer for both peptide series. This specific toxicity of the γ/γ-peptides towards *Leishmania* is a “must” for their use as CPP against the parasite; the deleterious effect of the doxorubicin on the parasite will be synergic with the leishmanicidal activity of the peptidic vehicle on its own. The tetradecameric Dox-γ-CC conjugate delivered a significant and faster leishmanicidal blow than the free drug. In addition, their *Leishmania*/mammalian cell selectivity index was higher than that of TAT, opening new avenues for the implementation of CPP-based therapies against the parasite. The molecular modeling stresses that the adoption of a well-defined conformation for the tetradecamers is highly influenced by the choice of the diastereomeric unit, even when the impact of the conformation on the leishmanicidal activity is not dramatic. It must be kept in mind that the importance of the peptide conformation relies on that adopted when in contact with the membrane. This is a well-documented effect on natural α-helical antimicrobial peptides, which are mostly devoid of a defined structure in solution, but are capable of forming a stable α-helix when in contact with the biological membranes or in membrane-mimicking solvents. Nevertheless, it must be highlighted that these γ/γ-peptides are much more conformationally restrained than those made of α-amino acids; hence, simulation with membranes will be performed in future. In our case, the CD results point to a defined conformational preference in solution of the free peptides. Moreover, an important fact from MD is that the doxorubicin moiety becomes wrapped by the peptide chain, regardless of the diastereomerism of the peptides employed, hidden from the aqueous environment while exposing the polar guanidinium groups at the periphery of the peptide, and improving the solubility of the drug.

## Supplementary Materials

The following are available online at https://www.mdpi.com/1422-0067/21/20/7502/s1, Scheme S1: Schematic protocol for the SPPS of the γ-CC and the γ-CT series; Table S1: General protocol for the solid phase synthesis of hybrid γ,γ-cyclobutane-proline peptides by Fmoc/Alloc strategy; Table S2: General protocol for the derivatization of the α-amine function using the solid phase synthesis; Table S3: General protocol for the incorporation of the 5(6)-carboxyfluorescein; Table S4: General protocol for the preparation of Tat_48-57_ and Tat_48-57_-CF; Table S5: Protocol for the additional Cys residue elongation of the peptides; Table S6: Peptide specifications for MD; Chart S1: Protecting groups used in the synthesis of TAT_48-57_; Figure S1: CD spectra (mean residual molar ellipticities) of peptides γ-CC **5** and γ-CT **9**; Figure S2: Main folding states identified during the simulation of: a) γ-CC **5** and b) γ-CT **9** isomers; Figure S3: Main folding states identified during the simulation of epimeric conjugates: a) (*R*)-Dox-γ-CC, (*R*)-**15** and b) (*S*)-Dox-γ-CC, (*S*)-**15**; Figure S4: Main folding states identified during the simulation of epimeric conjugates: a) (*R*)-Dox-γ-CT, (*R*)-**16** and b) (*S*)-Dox-γ-CT, (*S*)-**16**; Figure S5: Main folding states identified during the simulation of isomers: a) CF-γ-CC **7** and b) CF-γ-CT **11**; Figure S6: Representative MD conformation of conjugate (*S*)-Dox-γ-CC, (*S*)-**15**; Figure S7: Hydrogen bonding pattern as predicted by MD simulations for a) γ-CC **5**, b) (*R*)-Dox-γ-CC, (*R*)-**15**, and c) (*S*)-Dox-γ-CC, (*S*)-**15**; Figure S8: a) Hairpin conformation, and b) hydrogen bonding pattern as predicted by MD simulations for CF-CC **7**; Figure S9: a) Laminar conformation, and b) and c) hydrogen bonding pattern as predicted by MD simulations for CF-CC **7**; Figure S10: Hydrogen bonding pattern suggested by MD simulations for γ-CT **9**; Figure S11: a) Helical conformation for (*S*)-Dox-CT, (*S*)-**16**, and MD predicted hydrogen bonding pattern for b) (*R*)-Dox-CT, (*R*)-**16**, and c) (*S*)-Dox-CT, (*S*)-**16**; Figure S12: a) Conformation, and b) and c) hydrogen bonding pattern as predicted by MD simulations for CF-CT **11**; Synthesis and ^1^H and ^13^C NMR spectra of the new monomers; SPPS peptide synthesis procedures; cellular internalization of carboxyfluoresceinated peptides **6**, **7**, **10, 11** and TAT-CF in HeLa cells; CD spectra of γ-CC **5** and γ-CT **9**, main folding state MD studies; hydrogen bonding patterns predicted by MD simulations for γ-CC **5,** γ-CT **9,** Dox-γ-CC **15** and Dox-γ-CT **16**; conformations and associated hydrogen bonding patterns predicted by MD simulations for CF-γ-CC **7** and CF-γ-CT **11**; HPLC chromatograms and mass spectra of the purified peptides, and CF- and Dox-conjugates

## Abbreviations

ACN	Acetonitrile
Alloc	Allyloxycarbonyl
CBAA	Cyclobutane Amino Acid
CF	5(6)-Carboxyfluorescein
CPP	Cell Penetrating Peptide
DAPI	4′,6-Diamidino-2-phenylindole
DCM	Dichloromethane
DDS	Drug Delivery Systems
DFT	Density Functional Theory
DIPEA	Diisopropylethylamine
DMF	Dimethylformamide
DMSO	Dimethyl Sulfoxide
Dox	Doxorubicin
EDTA	Ethylenediaminetetraacetic Acid
ESI	Electrospray Ionization
FBS	Fetal Bovine Serum
Glc	Glucose
HBSS	Hanks Buffered Saline Solution
Fmoc	Fluorenylmethyloxycarbonyl
HPLC	High-Performance Liquid Chromatography
LPG	Lipophosphoglycan
MCC	4-(*N*-maleimidomethyl)cyclohexane-1-carboxylate
MD	Molecular Dynamics
MEM	Minimum Essential Medium
MTT	3-(4,5-Dimethylthiazol-2-yl)-2,5-diphenyltetrazolium Bromide
NMR	Nuclear Magnetic Resonance
PBS	Phosphate-Buffered Saline
PCA	Principal Component Analysis
PES	Potential Energy Surface
PI	Propidium Iodide
RESP	Restrained Electrostatic Potential
RMSD	Root-Mean-Square Deviation
RMSF	Root-Mean-Square Fluctuation
RP	Reverse Phase
SD	Standard Deviation
SM	Supplementary Materials
SMCC	Succinimidyl-4-(*N*-maleimidomethyl)cyclohexane-1-carboxylate
SPPS	Solid Phase Peptide Synthesis
