# Supplementary material for: Chiral Cyclobutane-Containing Cell-Penetrating Peptides as Selective Vectors for Anti-Leishmania Drug Delivery Systems
Source: Int J Mol Sci. 2020 Oct 12;21(20):7502. doi: 10.3390/ijms21207502 (PMC7590151; doi:10.3390/ijms21207502)
Supplement: Supplementary file 1 [file ijms-21-07502-s001.pdf]

SUPPLEMENTARY MATERIALS

Chiral Cyclobutane-Containing Cell-Penetrating Peptides as Selective Vectors for Anti-*Leishmania* Drug Delivery Systems

Ona Illa, José-Antonio Olivares, Nerea Gaztelumendi, Laura Martínez-Castro, Jimena Ospina, María-Ángeles Abengozar, Giuseppe Sciortino, Jean-Didier Maréchal, Carme Nogués*, Míriam Royo, Luis Rivas*, Rosa M. Ortuño*

Table of contents

Synthesis and ^{1}H and ^{13}C NMR spectra of the new monomers	S2
SPPS procedures	S6
CD Spectra of peptides γ-CC **5** and γ-CT 9	S13
Molecular Modeling studies	S14
HPLC and MS spectra of the purified peptides, and CF- and Dox-conjugates	S21
Abbreviations and References	S37

SYNTHESIS AND NMR SPECTRA OF THE NEW MONOMERS

(2*S*,4*R*)-4-(9H-fluoren-9-ylmethoxycarbonylamino)-1-(allyloxycarbonyl)-pyrrolidine-2-carboxylic acid

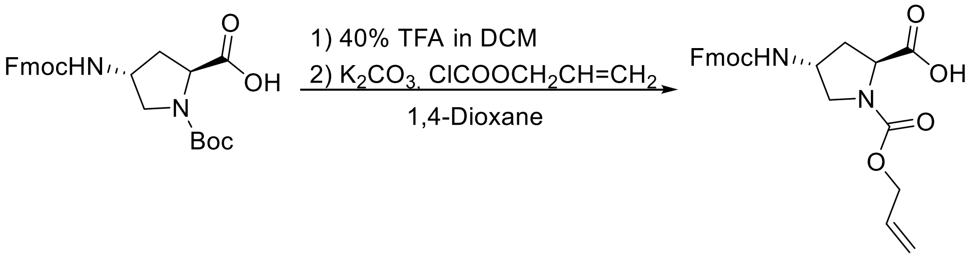

Commercially available (2*S*,4*R*)-4-(9H-fluoren-9-ylmethoxycarbonylamino)-1-(*tert*-butoxycarbonyl)pyrrolidine-2-carboxylic acid (2.00 g, 4.42 mmol) was dissolved in a 40% solution of TFA in dichloromethane (25 mL). The resulting mixture was stirred at room temperature for 30 minutes. The solvent and excess volatiles were evaporated under vacuum (coevaporations with 2 × 10 mL of DCM and 1 × 10 mL Et_2O). The intermediate ammonium salt (2.06 g, 4.42 mmol, quantitative yield) was obtained as a white solid and used without further purification. Then, the ammonium salt (2.06 g, 4.42 mmol) was dissolved in dioxane (20 mL). 25% K_2CO_3 aqueous solution (48 mL) and allyl chloroformate (0.56 mL, 5.30 mmol) were added. The reaction mixture was stirred at room temperature for 2 hours. The reaction mixture was acidified with 2 M HCl and extracted with EtOAc (3 x 30 mL). The organic layers were combined, dried over $MgSO_4$ and the solvent was evaporated under vacuum. The desired product (1.73 g, 3.96 mmol, 90% yield) was obtained as a white solid. $[\alpha]_D$: -13.8 (*c* = 1.1, MeOH); m.p.: 65-68 ^oC (EtOAc); IR (ATR): ν 3308, 2949, 1685, 1525 cm^{-1}; ^1H NMR (360 MHz, $CDCl_3$) δ 2.18-2.41 (m, 2H)3.42 (m, 1H), 3.84 (m, 1H), 4.20 (m, 1H), 4.36-4.63 (m, 6H), 5.26 (m, 2H, CH=C*H*$_2$), 5.89 (m, 1H, C*H*=CH_2), 6.23 (broad s, 1H, NH), 7.33 (t, *J* = 7Hz, 2H, H_{Fmoc}), 7.42 (t, *J* = 7Hz, 2H, H_{Fmoc}), 7.58 (d, *J* = 7 Hz, 2H, H_{Fmoc}), 7.78 (d, *J* = 7 Hz, 2H, H_{Fmoc}) ppm; ^{13}C NMR (100.6 MHz, $CDCl_3$) δ 35.2, 47.1, 50.0, 51.6, 57.8, 66.8, 118.1, 120.1, 124.9, 127.8, 132.2, 141.3, 143.7, 155.5, 156.1, 174.9 ppm; HRMS: Calculated for $C_{24}H_{23}N_2O_6$ $[M-H]^-$: 435.1551; Experimental: 435.1545.

(2*S*,4*S*)-4-(9H-fluoren-9-ylmethoxycarbonylamino)-1-(allyloxycarbonyl)-pyrrolidine-2-carboxylic acid

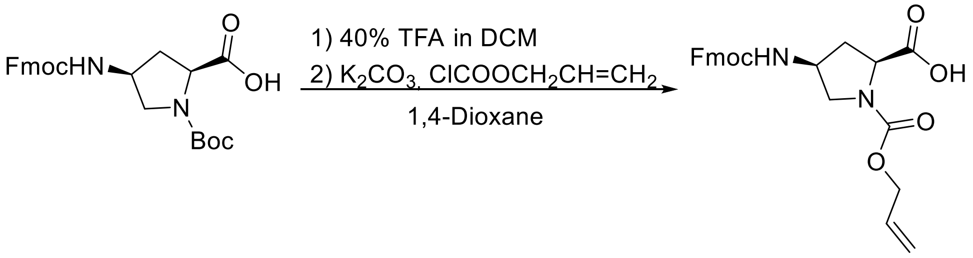

Commercially available (2*S*,4*S*)-4-(9H-fluoren-9-ylmethoxycarbonylamino)-1-(*tert*-butoxycarbonyl)pyrrolidine-2-carboxylic acid (2.11 g, 4.68 mmol) was dissolved in a 40% solution of TFA in dichloromethane (25 mL). The resulting mixture was stirred at room temperature for 30 minutes. The solvent and excess volatiles were evaporated under vacuum (coevaporations with 2 × 10 mL of DCM and 1 × 10 mL Et_2O). The intermediate ammonium salt (1.54 g, 4.68 mmol, quantitative yield) was obtained as a white solid which was used without further purification. Then, the ammonium salt (1.9 g, 5.39 mmol) was dissolved in dioxane (22 mL). 25% K_2CO_3 aqueous solution (56 mL) and allyl chloroformate (0.71 mL, 6.65 mmol) were added. The reaction mixture was stirred at room temperature for 2 hours. The reaction mixture was acidified with 2 M HCl and extracted with EtOAc (3 x 30 mL). The organic layers were combined, dried over $MgSO_4$ and the solvent was evaporated under vacuum. The desired product (1.84 g, 4.21 mmol, 90% yield) was obtained as a white solid. $[\alpha]_D$: -14.2 (*c* = 1.0, MeOH); m.p.: 70-73 ^oC (EtOAc); IR (ATR): ν 3304, 2945,1679, 1534 cm^{-1}; ^1H NMR (250 MHz, $CDCl_3$) δ 2.40 (m, 2H), 3.68 (m, 2H), 4.21 (m, 1H), 4.33-4.68 (m, 6H), 5.31 (m, 2H, CH=C*H*$_2$), 5.71 (m, 1H, NH), 5.93 (m, 1H, C*H*=CH_2), 7.33 (t, *J* = 7 Hz, 2H, H_{Fmoc}), 7.41 (t, *J* = 7 Hz, 2H, H_{Fmoc}), 7.58 (m, 2H, H_{Fmoc}), 7.77 (d, *J* = 7 Hz, 2H, H_{Fmoc}) ppm; ^{13}C NMR (100 MHz, $CDCl_3$) 34.6, 47.1, 50.5, 53.1, 57.4, 58.4, 67.0, 118.3, 120.0, 124.6, 125.1, 127.1, 127.8, 132.2, 141.3, 143.8, 154.4, 156.0, 175.4; HRMS: Calculated for $C_{24}H_{24}N_2NaO_6$ $[M+Na]^+$: 459.1527; Experimental: 459.1517.

^{1}H NMR (360 MHz, $CDCl_3$)

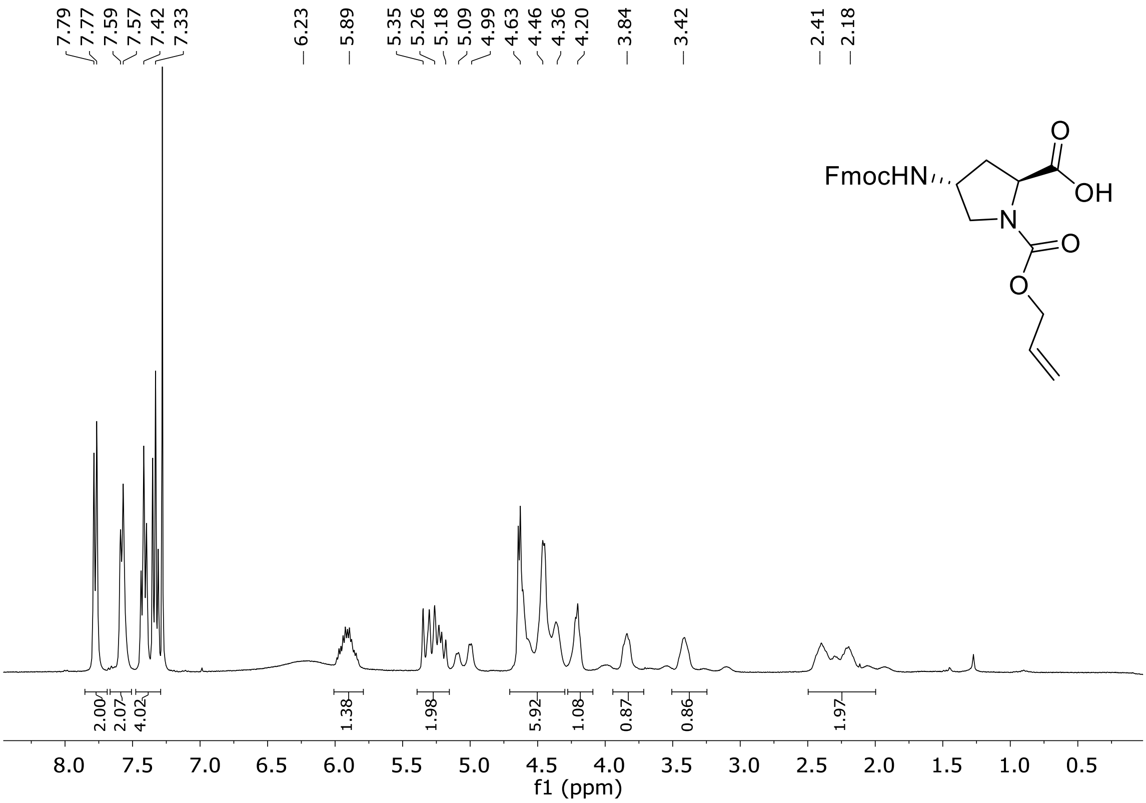

^{13}C NMR (100 MHz, $CDCl_3$)

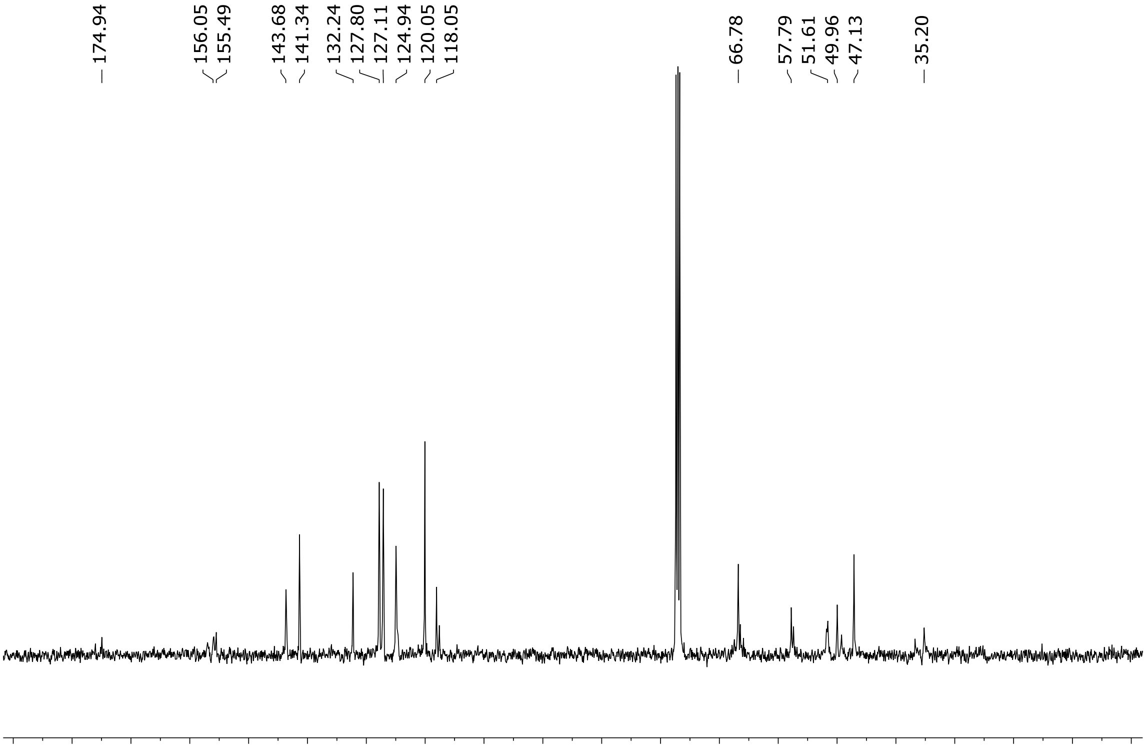

^{1}H NMR (360 MHz, $CDCl_3$)

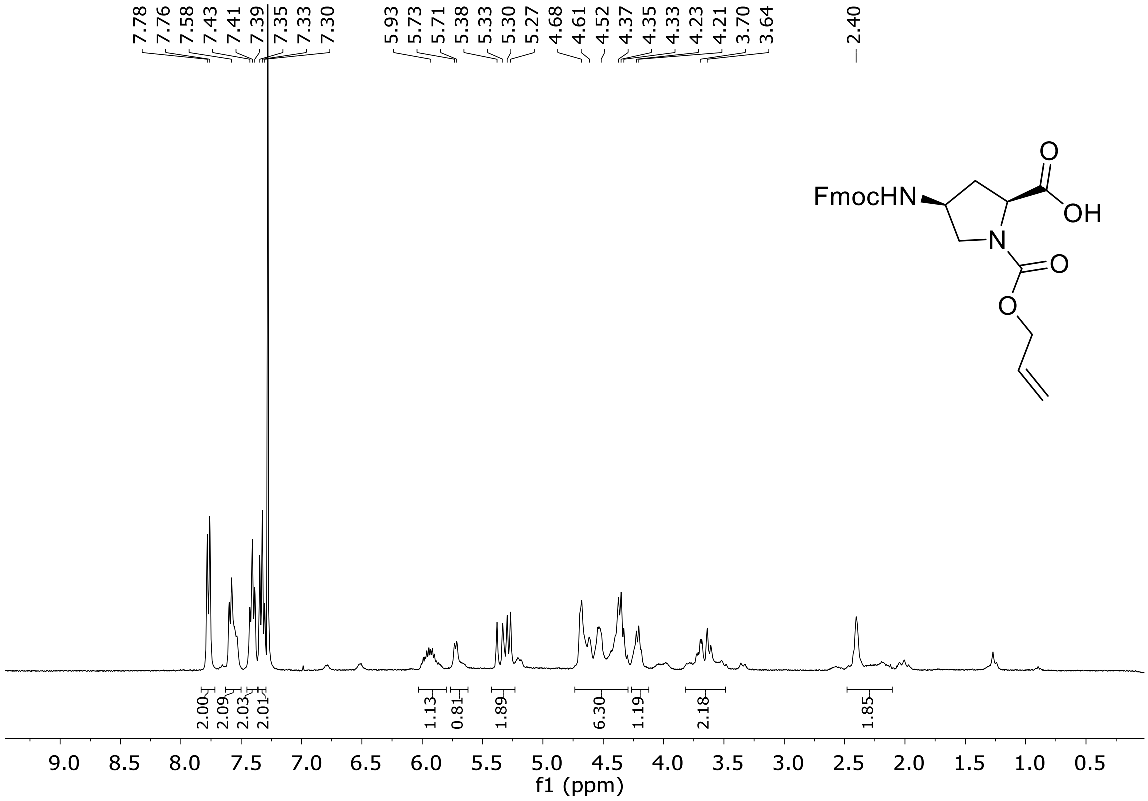

^{13}C NMR (100 MHz, $CDCl_3$)

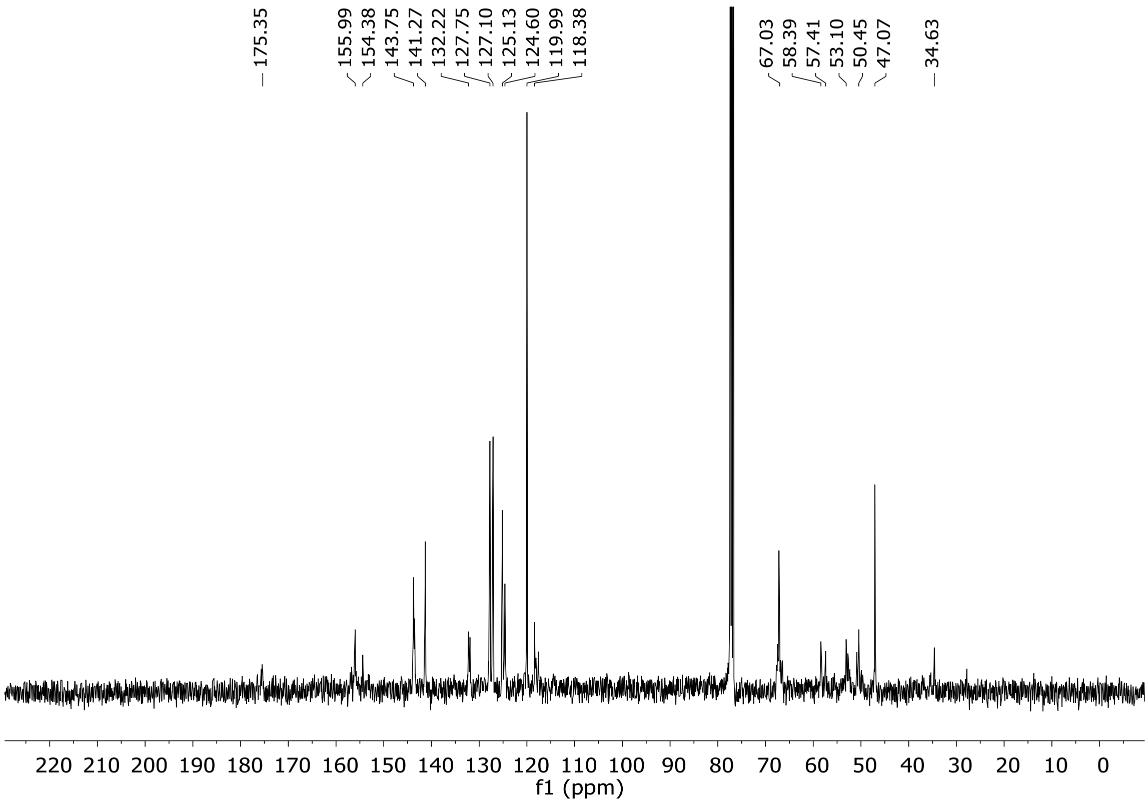

SOLID PHASE PEPTIDE SYNTHESIS PROCEDURES: γ-CC and γ-CT SERIES

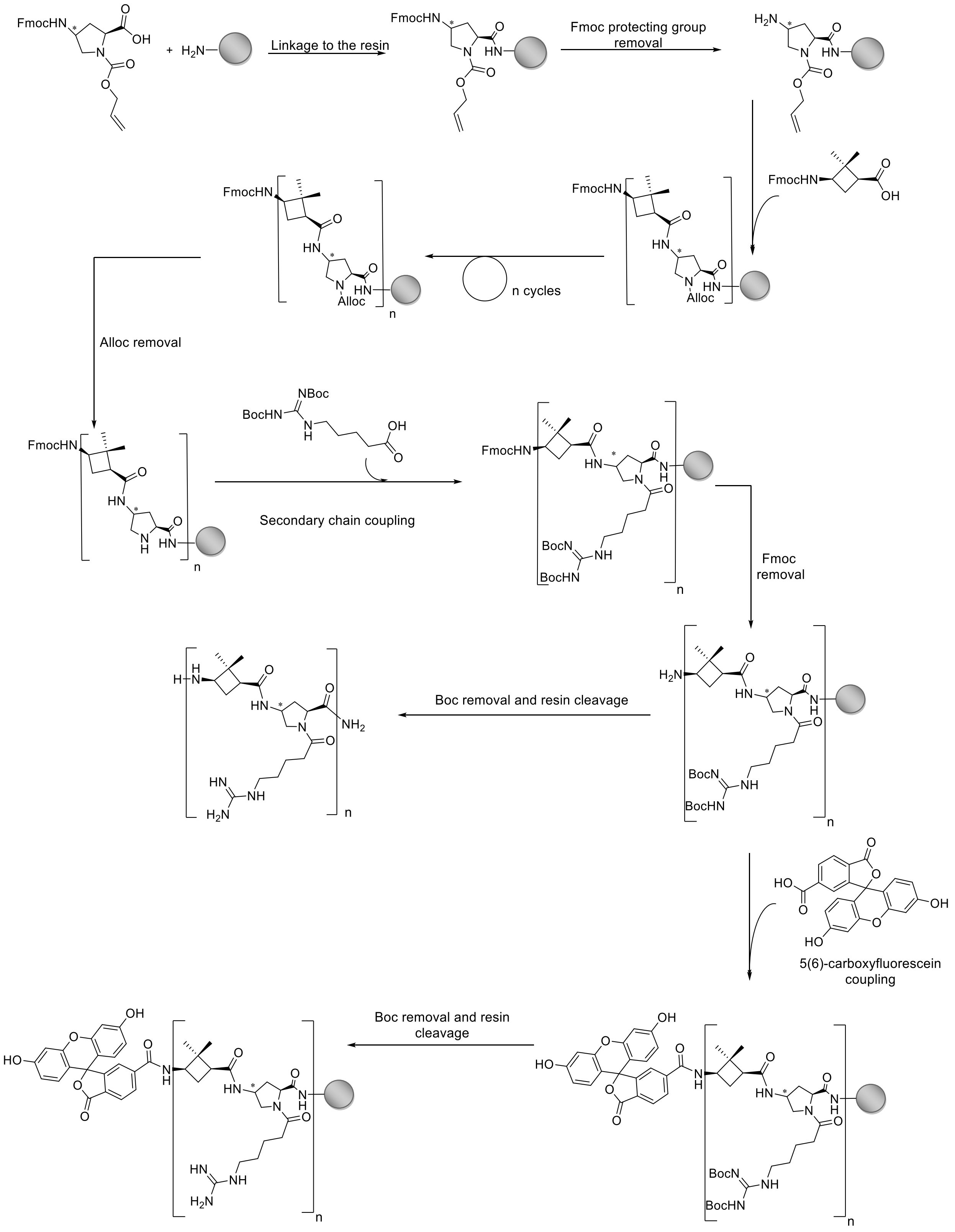

Scheme S1. Schematic protocol for the SPPS of the γ-CC and the γ-CT series.

Resin H-rink amide ChemMatrix® with 0.47 mmol/g functionalization was used, previously conditioned by several washes with DMF and DCM. The synthesis of both hybrid peptides and TAT_{48-57} was performed by using the Fmoc/Alloc strategy, although monomers for TAT_{48-57} contained other protecting groups. The protocol is summarized in **Table S1**.

Table S1. General protocol for the solid phase synthesis of Hybrid γ,γ-cyclobutane-proline peptides, by Fmoc/Alloc strategy.

Step	Reagents /Solvents	Aim	Cycles	t/cycle (min)
1	DCM	Wash	5	1
2	DMF	Wash	5	1
3	*cis*- or *trans*-Fmoc-γ-amino-L-proline/DIC/OxymaPure® (2.5:2.5:2.5) in DMF	Coupling	1	120
4	DMF	Wash	5	1
5	DCM	Wash	5	1
6	Ninhydrine test (-)	Coupling test	1	3
7	Piperidine/DMF (2:8, v/v)	Deprotection	3	10
8	DMF	Wash	5	1
9	DCM	Wash	5	1
10	Ninhydrine test (+)	Deprotection test	1	3
11	DMF	Wash	5	1
12	(1*S*,3*R*)-Fmoc-γ-amino-cyclobutane amino acid/OxymaPure®/PyBOP/DIPEA (2.5:2.5:2.5:5) in DMF	Coupling	1	120
13	DMF	Wash	5	1
14	DCM	Wash	5	1
15	Ninhydrine test (-)	Coupling test	1	3
16	Piperidine/DMF (2:8, v/v)	Wash	3	10
17	DMF	Wash	5	1
18	DCM	Wash	5	1
19	Ninhydrine test (+)	Deprotection test	1	3

Steps from 1 to 19 were repeated n (n = 6 or 7) times to obtain the dodecamer or tetradecamer peptides, respectively. By the time the desired peptide was obtained, 200 mg of resin were separated for further reactions.

Once the peptide skeleton was prepared, the derivatization of the α-amino function was performed. In a first step, the Alloc protecting groups were removed by catalytic reduction using palladium, and then the guanidinylated lateral chain (5-(2,3-bis(*tert*-butoxycarbonyl)guanidino)pentanoic acid), previously synthesized in solution,[S1] was incorporated using OxymaPure® as coupling agent. After that, the Fmoc group of the terminal residue was removed (**Table S2**).

Table S2. General protocol for the derivatization of the α-amine function using the solid phase synthesis.

Step	Reagents /Solvents	Aim	Cycles	t/cycle (min)
1	DCM	Wash	5	1
2	$PhSiH_3/Pd(PPh_3)_4$ (12:0.1) in DCM	Deprotection	2	15
3	DCM	Wash	5	1
4	DMF	Wash	5	1
5	$(Et)_2NCSSNa\ 3H_2O$ (20 mM in DMF)	Palladium Wash	5	1
6	DMF	Wash	5	1
7	DCM	Wash	5	1
8	Chloranil (+)	Deprotection test	1	3
9	DMF	Wash	5	1
10	5-(2,3-bis(*tert*-butoxycarbonyl)guanidino)pentanoic acid/DIC/OxymaPure® (2.5:2.5:2.5) for each proline in DMF	Coupling	1	120
11	DMF	Wash	5	1
12	DCM	Wash	5	1
13	Chloranil (-)	Coupling test	1	3
14	Piperidine/DMF (2:8, v/v)	Deprotection	3	10
15	DMF	Wash	5	1
16	DCM	Wash	5	1
17	Ninhydrine test (+)	Deprotection test	1	3

Once the functionalization of the α-amino group was finished, the peptide resin (200 mg) was split into two equal parts. Half of it (100 mg) was used to obtain the free amine peptides, and the remaining resin was used for the incorporation of the carboxyfluorescein (CF) in the *N*-terminal group (**Table S3**).

Table S3. General protocol for the incorporation of the 5(6)-carboxyfluorescein.

Step	Reagents /Solvents	Aim	Cycles	t/cycle (min)
1	DMF	Wash	5	1
2	CF/ OxymaPure®/PyBOP/DIPEA (4:6:4:6) in DMF	Coupling	1	120
3	DMF	Wash	5	1
4	DCM	Wash	5	1
5	Ninhydrine test (-)	Deprotection test	1	3
6	TFA/(^{i}Pr)$_3$SiH/H_2O (95:2.5:2.5)	Deprotection / Resin cleavage	1	120
7	DCM	Wash	5	1

Cleaveage from the Aminomethyl-ChemMatrix® resin and removal of the Boc carbamate protecting groups: acid hydrolysis

The cleavage of the peptide from the resin was carried out by acid hydrolysis using a mixture of TFA/TIS/H_2O (95:2.5:2.5) for 3 h under stirring.

The peptide crude was separated from the resin through filtration. The solid was washed with DCM (4 ×). The solution was concentrated under vacuum but not until dryness. Then, the peptide was precipitated through addition of cold Et_2O. The solid was filtered and centrifuged with Et_2O (3 ×). The resulting solid was dissolved in ACN:H_2O (1:1, v/v) and lyophilized.

TAT$_{48-57}$ and TAT$_{48-57}$-CF SYNTHESIS

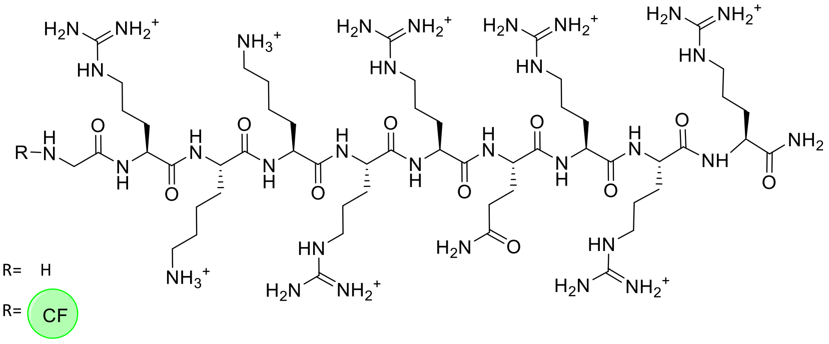

H-Rink amide-ChemMatrix® resin with 0.49 mmol/g functionalization was used. It was conditioned with successive washes with DMF and DCM. N^{α}– Fmoc protected amino acids were used for the synthesis. Protecting groups for side chains were Pbf (Arg), Trt (Gln), Boc (Lys). The protocol is summarized in **Table S4**.

Table S4. General protocol for the preparation of Tat$_{48-57}$ and Tat$_{48-57}$-CF

Step	Reagents /Solvents	Aim	Cycles	t/cycle (min)
1	DCM	Wash	5	1
2	DMF	Wash	5	1
3	Fmoc-L-Arg(Pbf)-OH(**R**)/OxymaPure®/DIC (3:3:3) in DMF	Coupling	1	120
4	DMF	Wash	5	1
5	DCM	Wash	5	1
6	Ninhydrine test (-)	Coupling test	1	3
7	Piperidine/DMF (2:8, v/v)	Deprotection	3	10
8	DMF	Wash	5	1
9	DCM	Wash	5	1
10	Ninhydrine test (+)	Deprotection test	1	3
11	DMF	Wash	5	1
12	Fmoc-L-Gln(Trt)-OH(**Q**)/OxymaPure®/ DIC (3:3:3) in DMF	Coupling	1	120
13	DMF	Wash	5	1
14	Fmoc-L-Lys(Boc)-OH(**K**)/OxymaPure®/ DIC (3:3:3) in DMF	Coupling	1	120
15	DMF	Wash	5	1
16	Fmoc-Gly-OH(**G**)/OxymaPure®/ DIC (3:3:3) in DMF	Coupling	1	120

Sequence: Steps 1-11 (twice), 1-13 (once), 5-10 (once), 2-10 (twice), 13-14 (once), 5-10 (once), 13-14 (once), 5-10 (once), 2-10 (once), 15-16 (once).

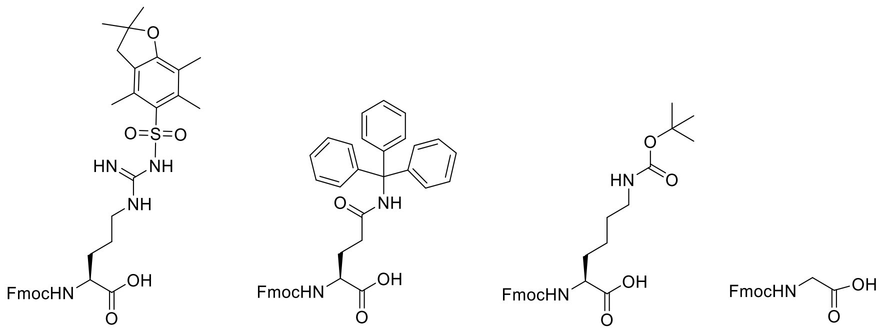

Arginine (Arg, R) Glutamine (Gln, Q) Lysine (Lys, K) Glycine (Gly, G)

Chart S1. Protecting groups used in the synthesis of $TAT_{48\text{-}57}$

Once the desired length peptide were prepared and functionalized, the resin (1 g) was split into two equal parts, and each of them used for the synthesis of the free amino peptide, or carboxyfluoresceinated (CF) at the *N*-terminal amino group (see Protocol in **Table S3**)

Cleavage from the H-Rink amide ChemMatrix®resin: The same protocol described above for the Aminomethyl-ChemMatrix® resin was used.

γ-CC / γ-CT / TAT$_{48-57}$ CONJUGATION WITH DOXORUBICIN

The peptides were synthesized according to the protocols described in Tables S1 and S2. Then, the primary chain was elongated with a Cys(Trt) residue, protected by an acetyl group (**Table S5**).

Table S5. Protocol for the additional Cys residue elongation of the peptides.

**Step**	**Reagents /Solvents**	**Aim**	**Cycles**	**t/cycle (min)**
1	DCM	Wash	5	1
2	DMF	Wash	5	1
3	Fmoc-L-Cys(Trt)-OH(**C**)/OxymaPure®/DIC (3:3:3) in DMF	Coupling	1	120
4	DMF	Wash	5	1
5	DCM	Wash	5	1
6	Ninhydrine test (-)	Coupling test	1	3
7	Piperidine/DMF (2:8, v/v)	Deprotection	3	10
8	DMF	Wash	5	1
9	DCM	Wash	5	1
10	Ninhydrine test (+)	Deprotection test	1	3
9	DMF	Wash	5	1
11	Ac_2O, DIPEA (5:5) in DMF	Protection of NH_2	1	100
13	DMF	Wash	5	1
14	DCM	Wash	5	1
15	Ninhydrine test (-)	Coupling test	1	3
16	Piperidine/DMF (2:8, v/v)	Wash	3	10
17	DMF	Wash	5	1
18	DCM	Wash	5	1
19	Ninhydrine test (+)	Deprotection test	1	3

Once the peptide was purified, it was coupled to Doxorubicin using the linker (SMCC), as described in the Experimental Section of the manuscript.

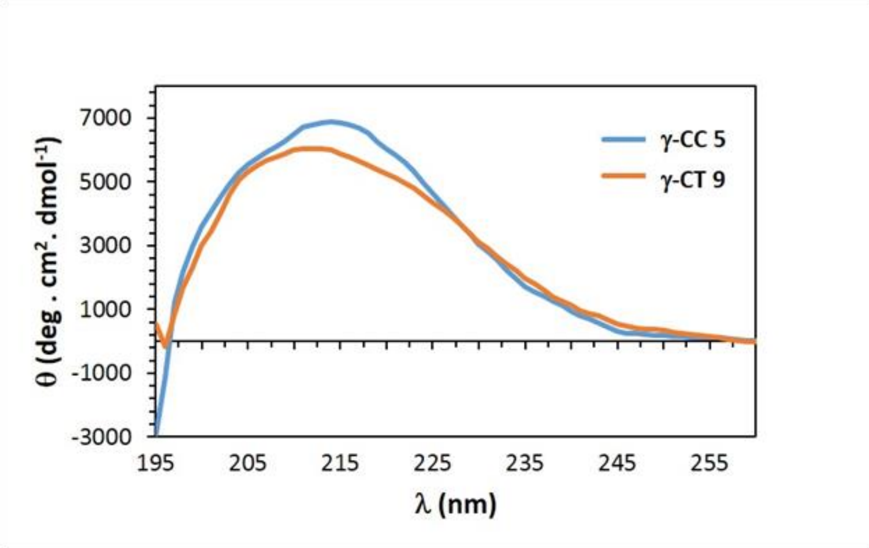

Figure S1. CD spectra (mean residual molar ellipticities) of peptides γ–CC **5** and γ–CT **9**. The spectra were recorded with 50 μM solutions in PBS.

MOLECULAR MODELING STUDIES

For all the MD simulations, 1000 energy minimization steps were carried out holding the peptide fixed in order to relax the water box. Subsequent 2500 energy minimization steps were performed to the whole system. Then, thermalization of water molecules while keeping the peptide fixed was achieved by increasing the temperature from 0 K to 300 Kand followed by a thermalization of the peptide at 300 K.

The strategy followed to perform the MD for the entire set of systems was the following: i) building the model for the peptides γ-CC **5** and γ-CT **9**, and ii) after identification of stable conformations, replace the *N*-terminal residues by carboxyfluorescein or doxorubicin, and run subsequent MDs

Table S6. Peptide specifications for MD

Peptide	MD time (ns)	Atoms	Water molecules	Counterions (Cl^-)
γ-CC **5**	200	411	10868	8
γ-CT **9**	200	411	9897	8
CF-γ-CC **7**	300	447	10232	7
CF-γ-CT **11**	450	447	5574	7
(*R*)-DOX-γ-CC (*R*)-**15**	300	519	7311	8
(*S*)-DOX-γ-CC (*S*)-**15**	300	519	5924	8
(*R*)-DOX-γ-CC (*R*)-**16**	400	519	7272	8
(*S*)-DOX-γ-CT (*S*)-**16**	300	519	7148	8

In order to determine the convergence of simulations and to ascertain wether the conformational space was sampled enough, trajectories were analyzed with CPPTraj tools of the AmberTools18 package.[2] The main indicator was the recurrence of a relative stable conformation (or several conformations) for a statistically relevant number of times. Root-mean-square deviation (RMSD) from those structures, all-to-all frames RMSD and counting cluster analyses were carried out considering backbone atoms (C^{α} in γ-proline and C^{γ} in the γ-CBAA) so as to avoid flexible moieties that might distort the results.

Furthermore, a principal component analysis (PCA) was performed to determine whether the dynamic transitions take place within distinct conformations.

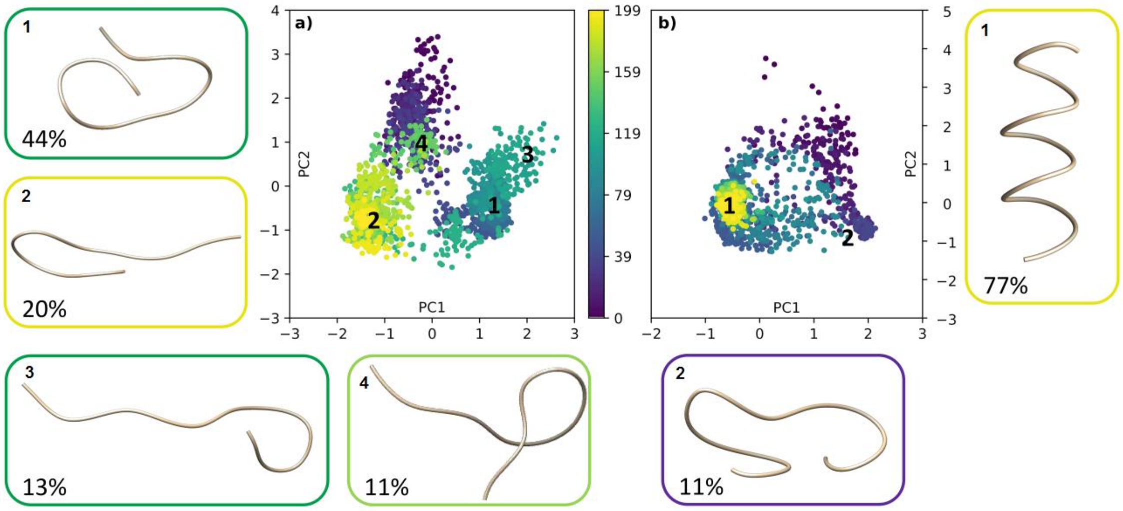

Figure S2. Main folding states identified during the simulation of: a) γ-CC **5** and b) γ-CT **9** isomers.

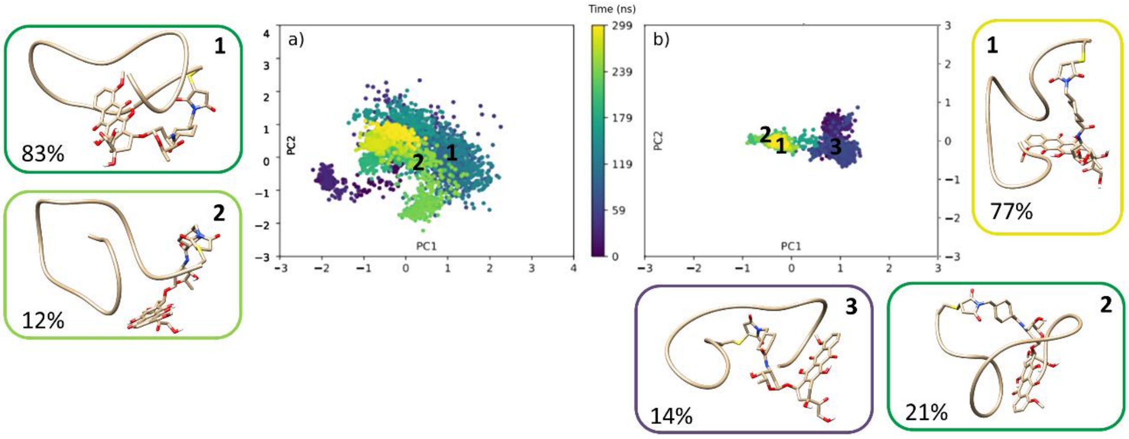

Figure S3. Main folding states identified during the simulation of epimeric conjugates: a) (*R*)-Dox-γ-CC, (*R*)-**15** and b) (*S*)-Dox-γ-CC, (*S*)-**15**

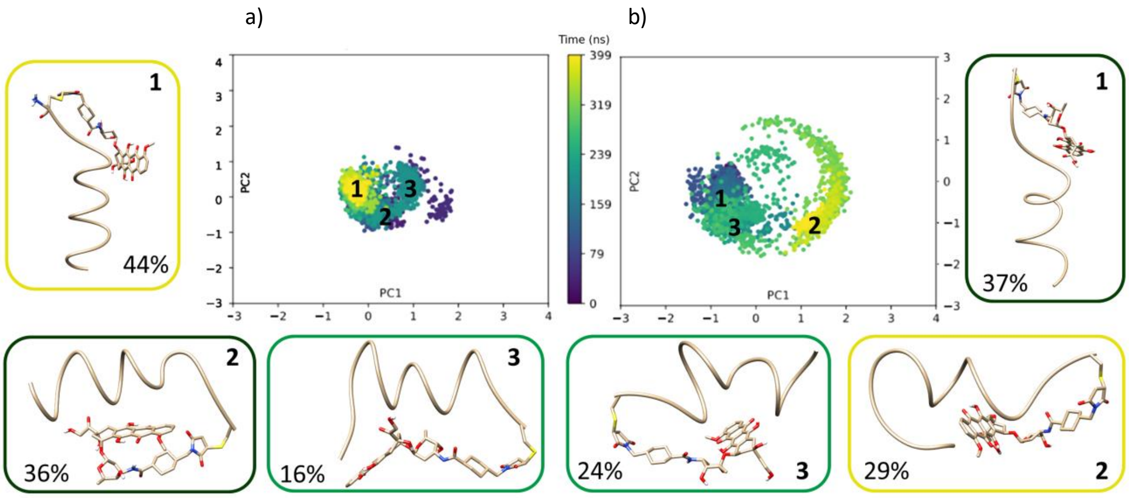

Figure S4. Main folding states identified during the simulation of epimeric conjugates: a) (*R*)-Dox-γ-CT, (*R*)-**16** and b) (*S*)-Dox-γ-CT, (*S*)-**16**

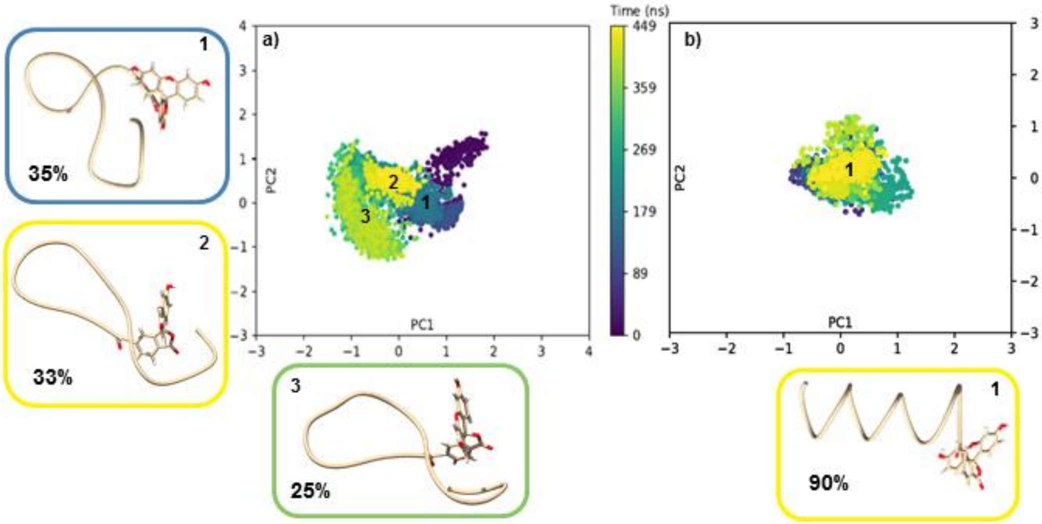

Figure S5. Main folding states identified during the simulation of isomers: a) CF-γ-CC **7** and b) CF-γ-CT **11**.

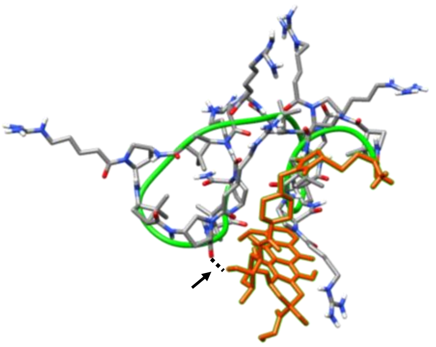

Figure S6. Representative MD conformation of conjugate (*S*)-Dox-γ-CC, (*S*)-**15**. The polypeptide scaffold is highlighted by the green ribbon; Dox is represented in orange; the arrow points out long-range hydrogen-bonding between the hydroxyl group of the amino sugar ring in Dox-moiety and proline residue *i* = 12 (See Figure S7).

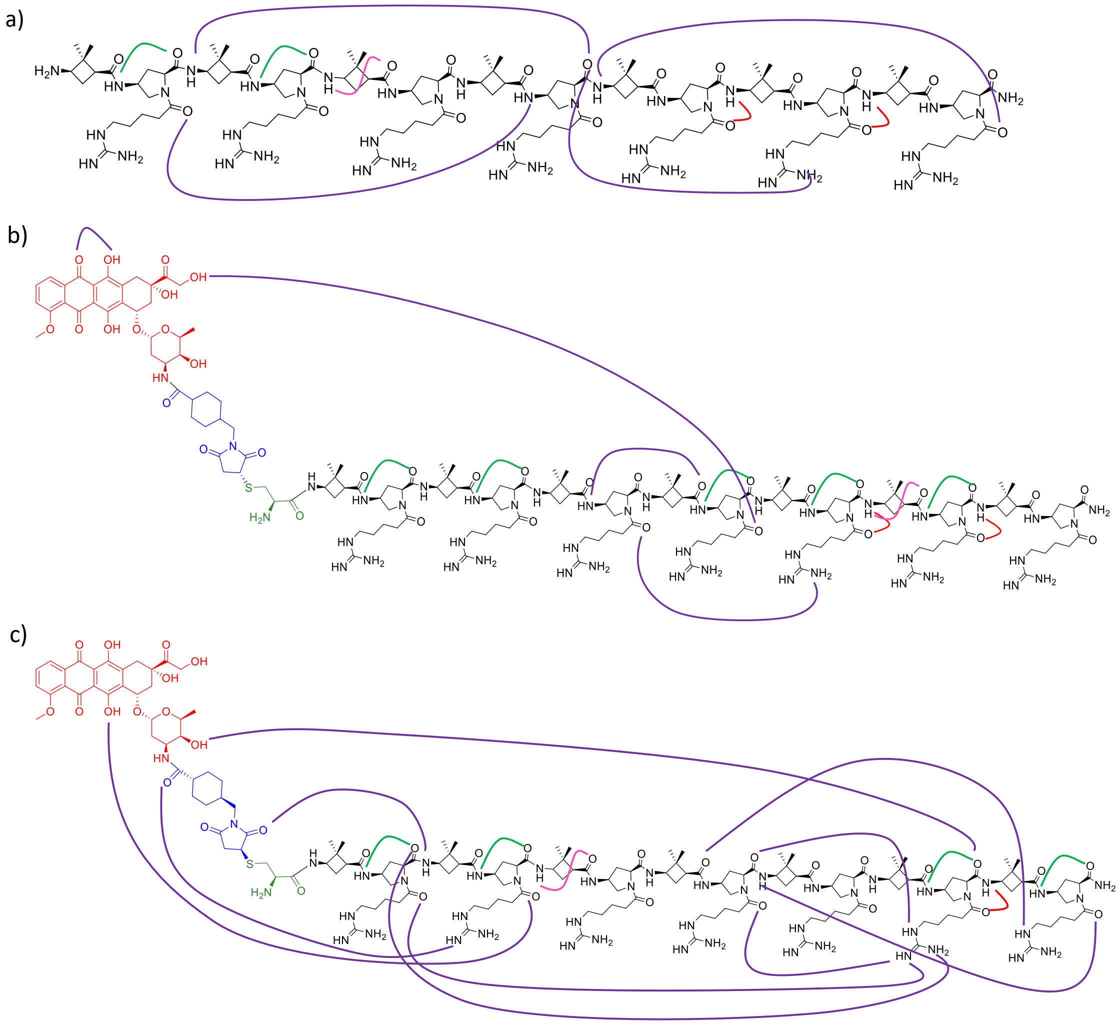

Figure S7. Hydrogen bonding pattern as predicted by MD simulations for a) γ-CC **5,** b) (*R*)-Dox-γ-CC, (*R*)-**15,** and c) (*S*)-Dox-γ-CC, (*S*)-**15**.

a)

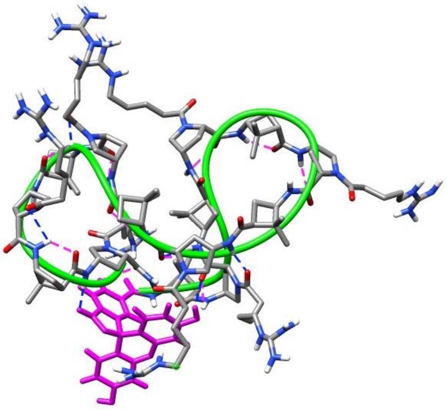

b)

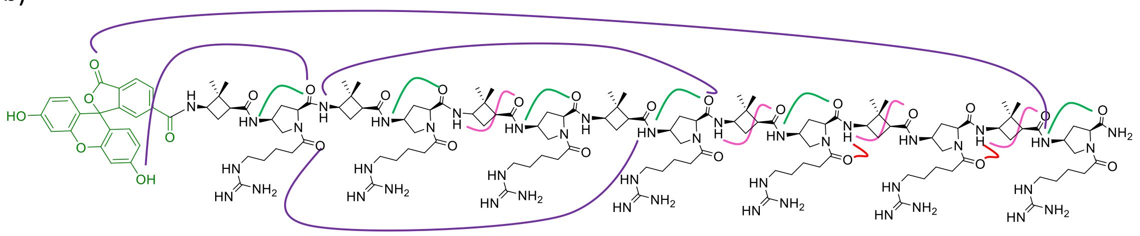

Figure S8. a) Hairpin conformation (peptide scaffold is highlighted by the green ribbon and CF is represented in magenta), and b) hydrogen bonding pattern as predicted by MD simulations for CF-CC **7**.

a)

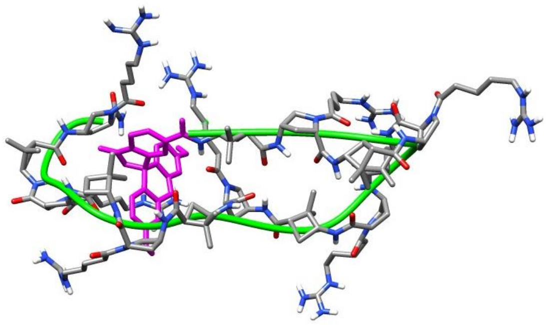

b)

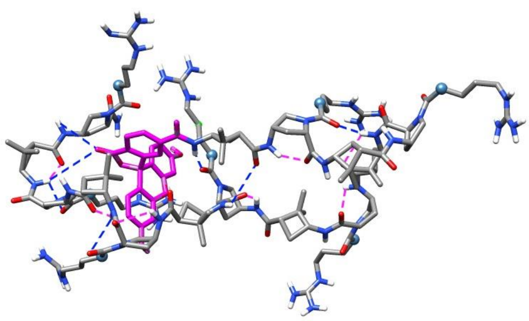

c)

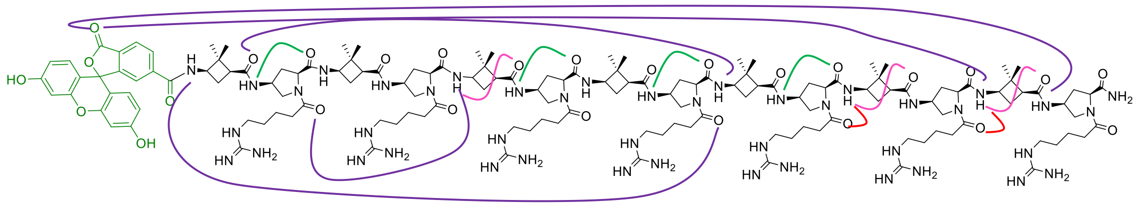

Figure S9. a) Laminar conformation (peptide scaffold is highlighted by the green ribbon and CF is represented in magenta), and b) and c) hydrogen bonding pattern as predicted by MD simulations for CF-CC **7**

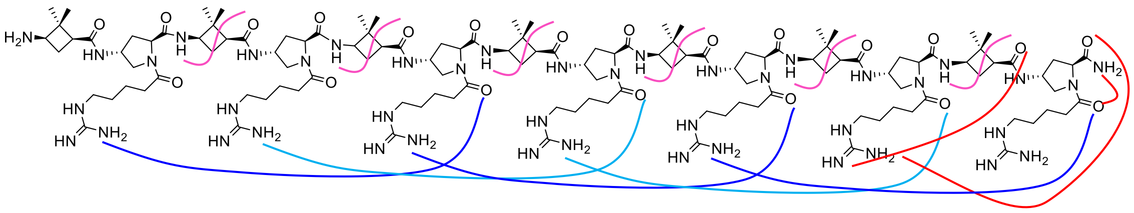

Figure S10. Hydrogen bonding pattern suggested by MD simulations for γ-CT **9**

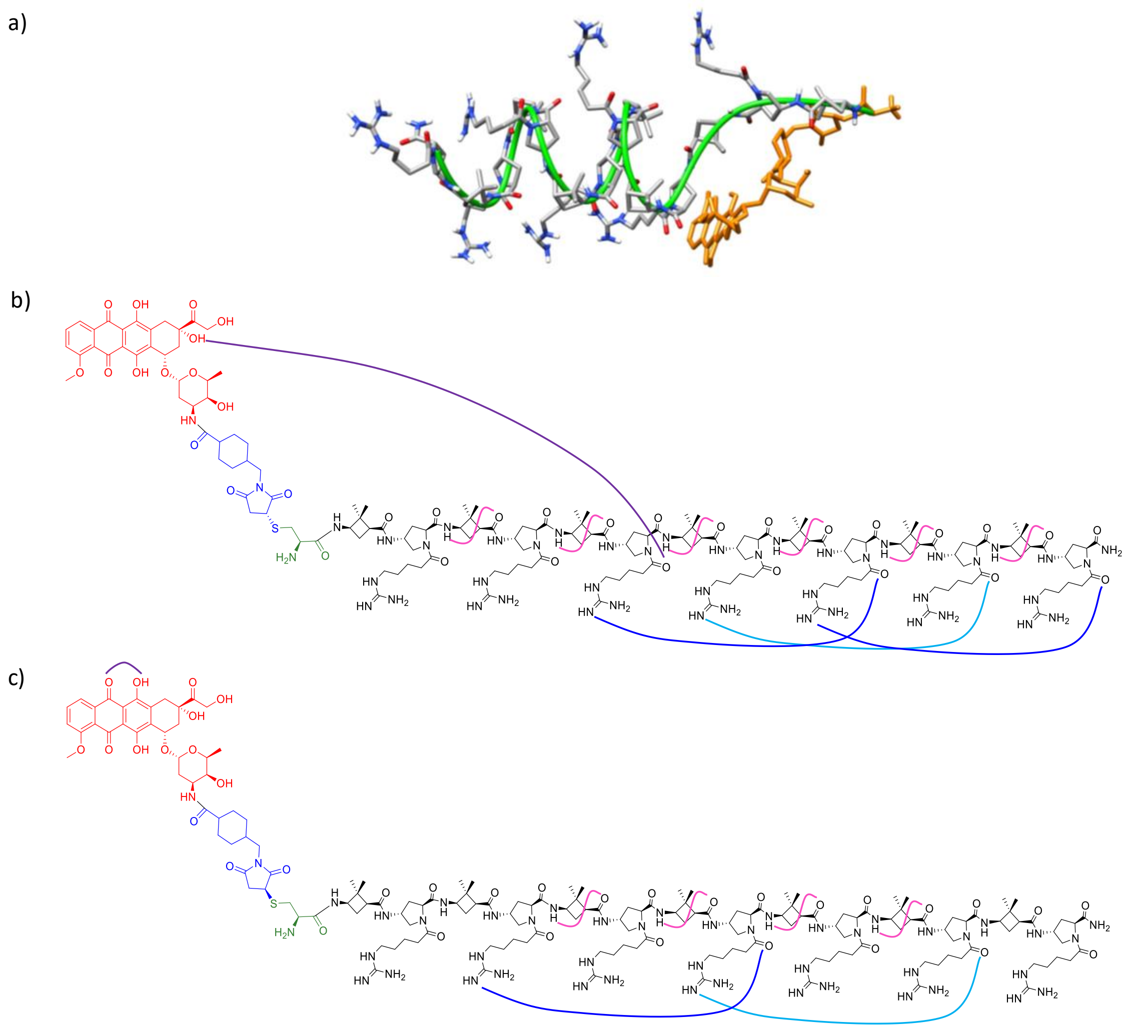

Figure S11. a) Helical conformation for (*S*)-Dox-CT, (*S*)-**16** (peptide scaffold is highlighted by the green ribbon and Dox is represented in orange), and MD predicted hydrogen bonding pattern for b) (*R*)-Dox-CT, (*R*)-**16**, and c) (*S*)-Dox-CT, (*S*)-**16**.

a)

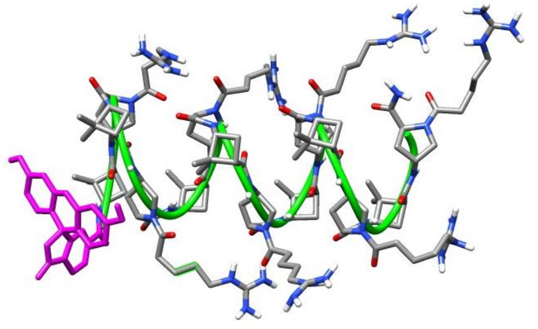

b)

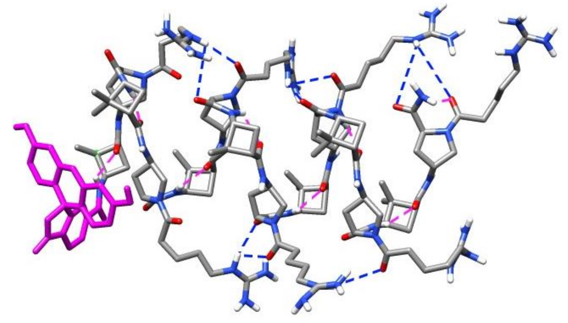

b)

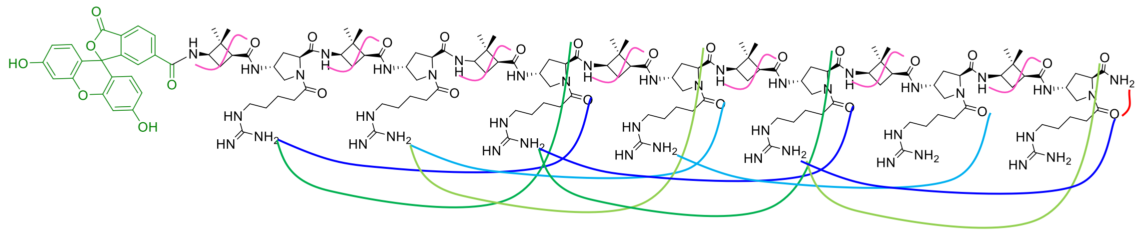

Figure S12. a) Conformation (peptide scaffold is highlighted by the green ribbon and CF is represented in magenta), and b) and c) hydrogen bonding pattern as predicted by MD simulations for CF-CT **11**.

HPLC CHROMATOGRAMS and MASS SPECTRA of the PURIFIED PEPTIDES, and CF- and Dox-CONJUGATES

TAT$_{48-57}$:

RP-HPLC

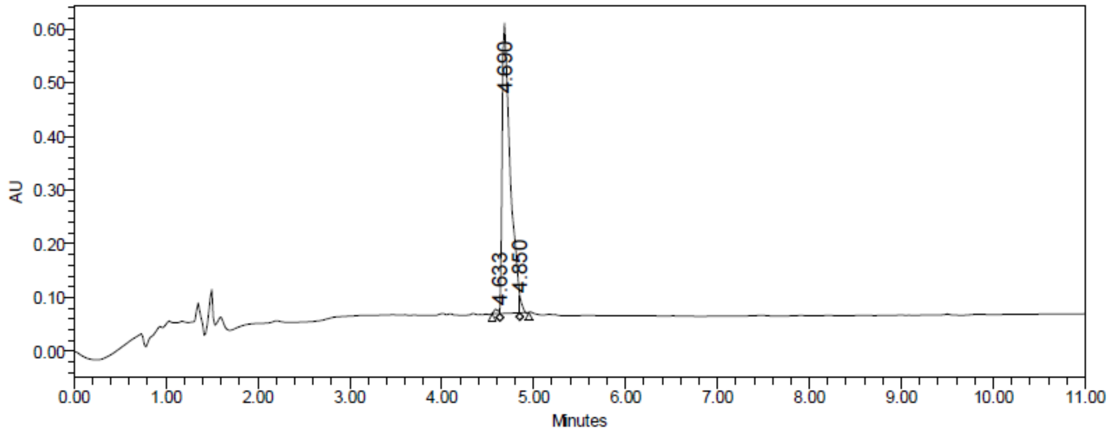

	RT	Area	% Area	Height
1	4.633	28955	0.87	9336
2	4.690	3255706	97.59	555018
3	4.850	51534	1.54	31400

m/z (ESI):

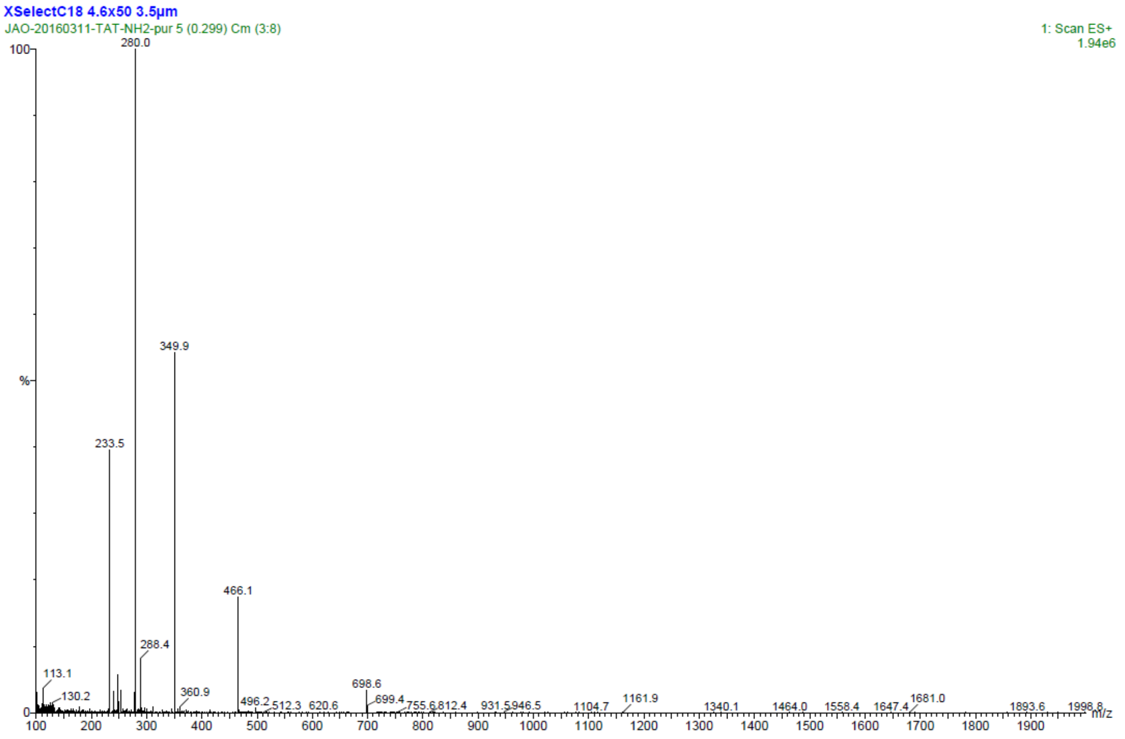

CF-TAT48-57:

RP-HPLC

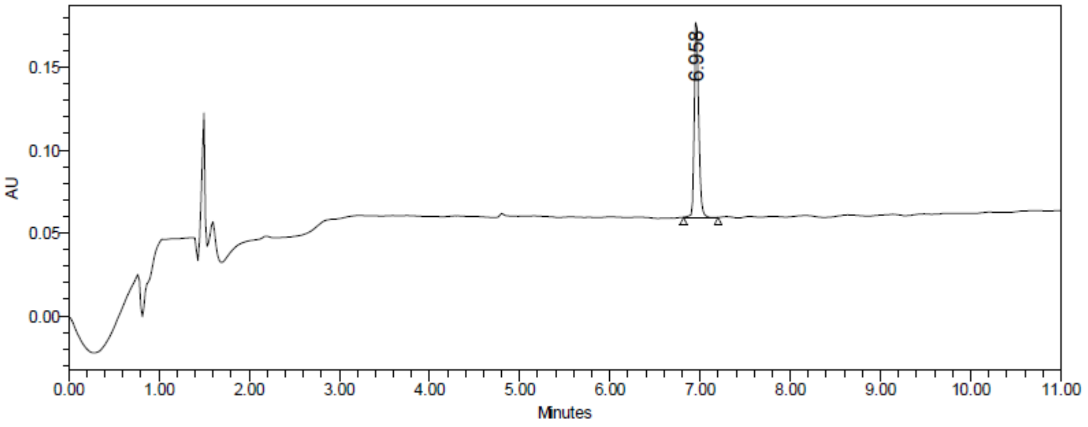

	RT	Area	% Area	Height
1	6.958	403011	100.00	111925

***m/z* (ESI):**

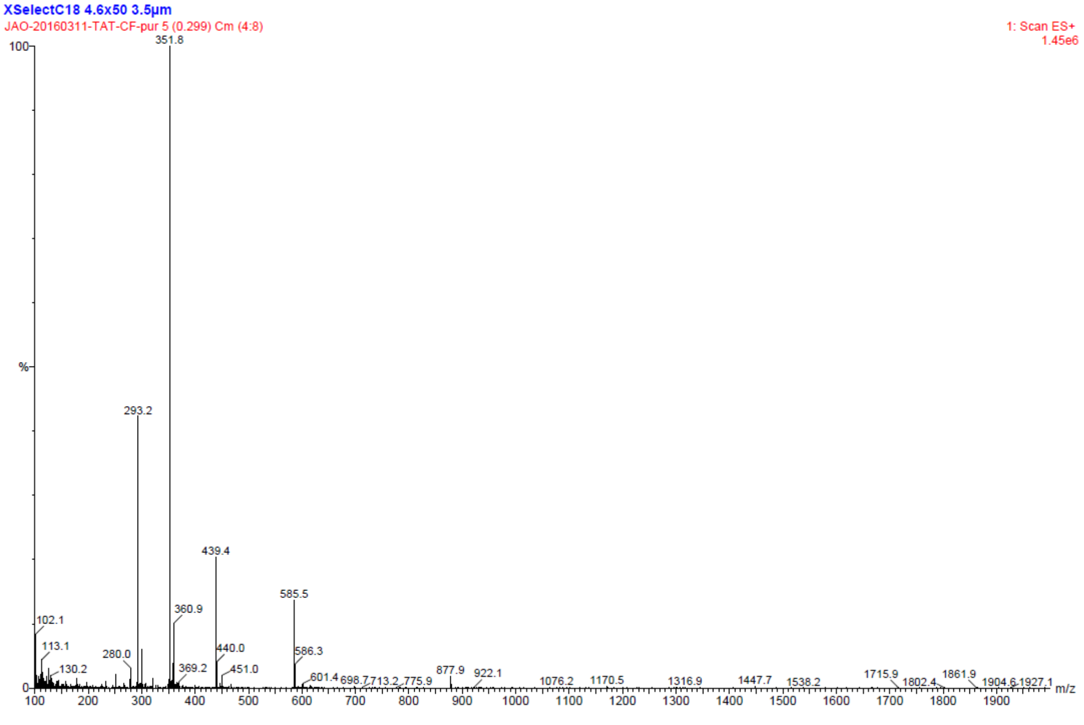

TAT$_{48-57}$-Cys:

RP-HPLC:

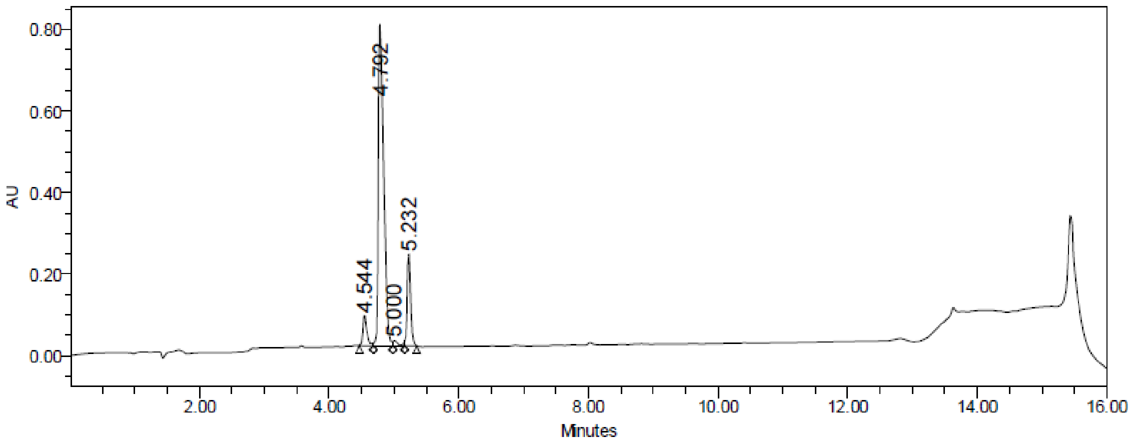

	RT	Area	% Area	Height
1	4.544	325668	5.76	70288
2	4.792	4438951	78.56	803772
3	5.000	71345	1.26	12066
4	5.232	814082	14.41	217480

***m/z* (ESI):**

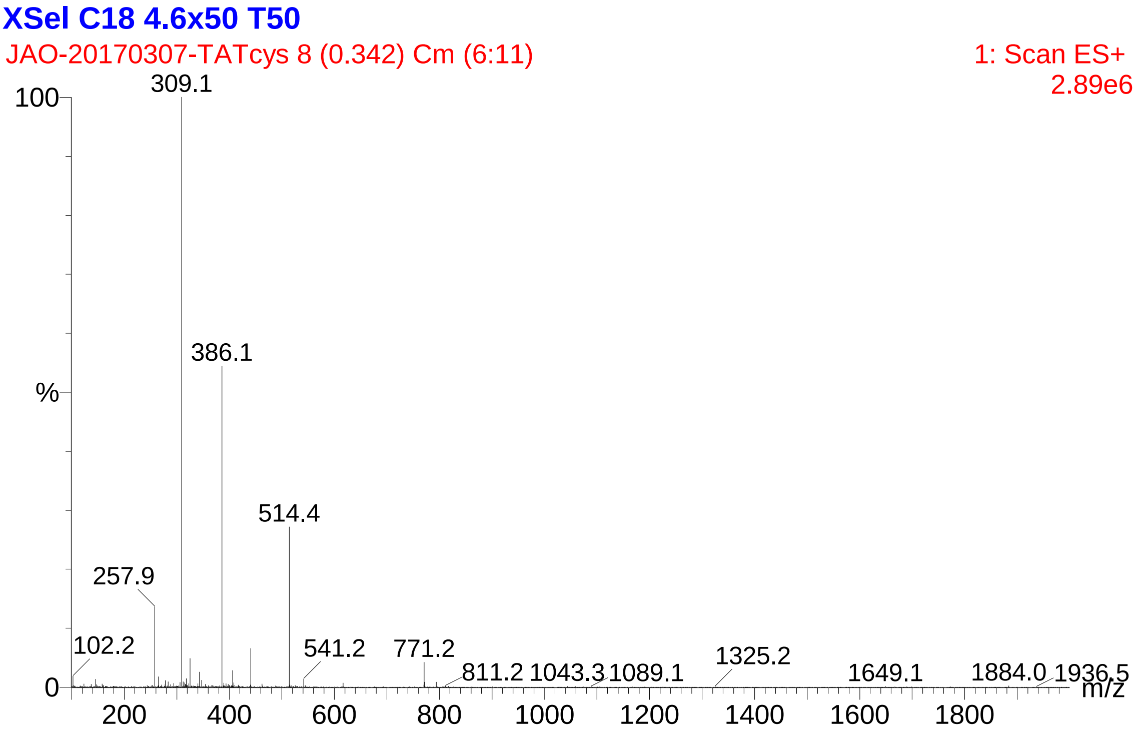

Dox-TAT$_{48-57}$:

RP-HPLC:

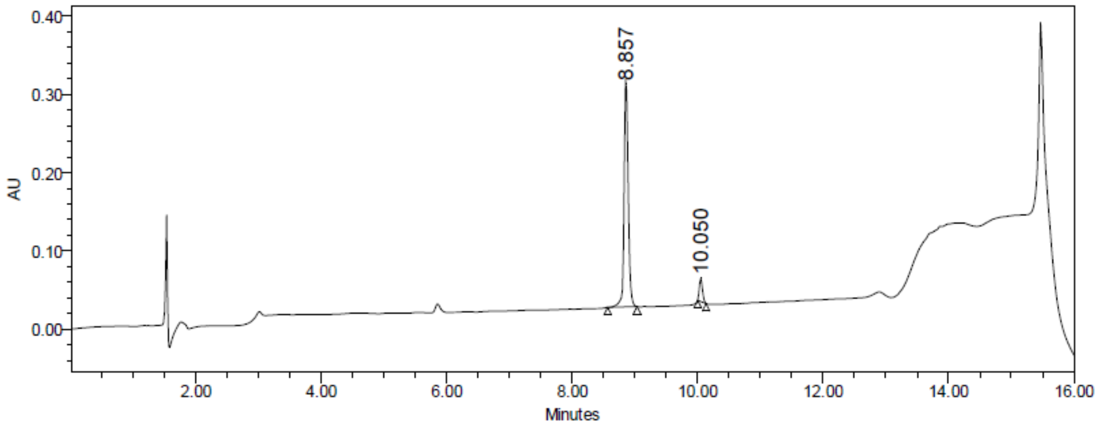

	RT	Area	% Area	Height
1	8.857	1324675	92.80	288149
2	8.857	1331092	92.83	288430
3	10.050	102787	7.20	28710
4	10.050	102787	7.17	28710

m/z (ESI):

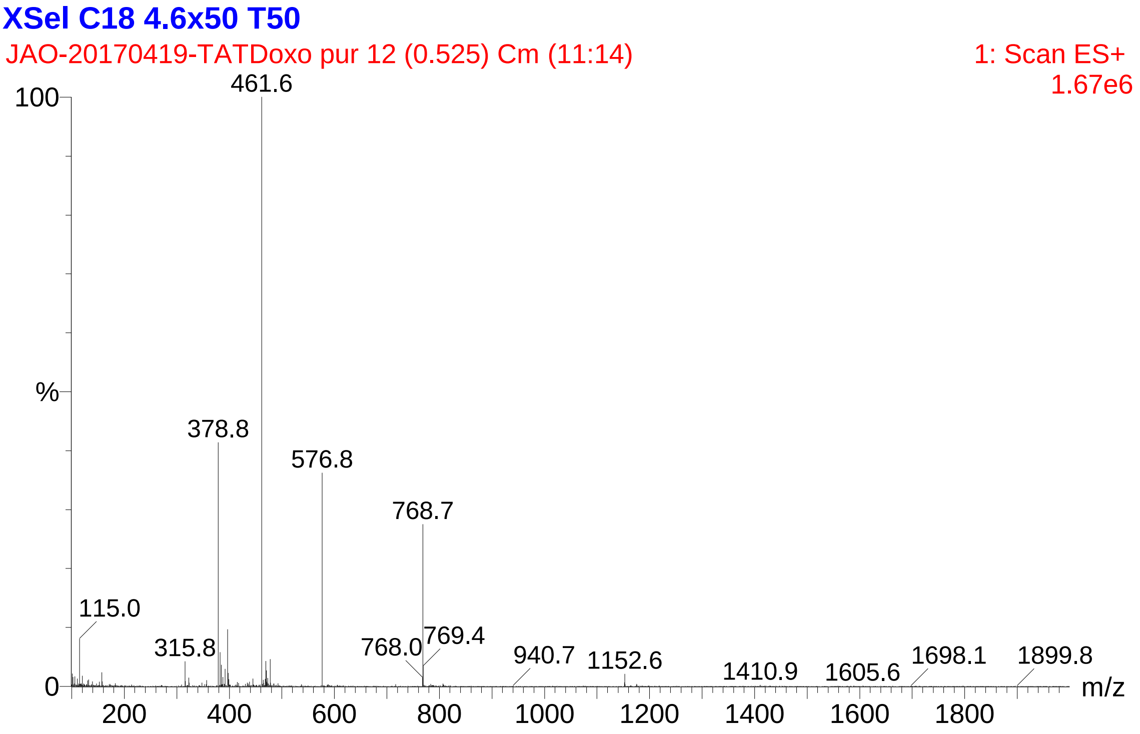

Peptide γ-CC 4:

RP-HPLC:

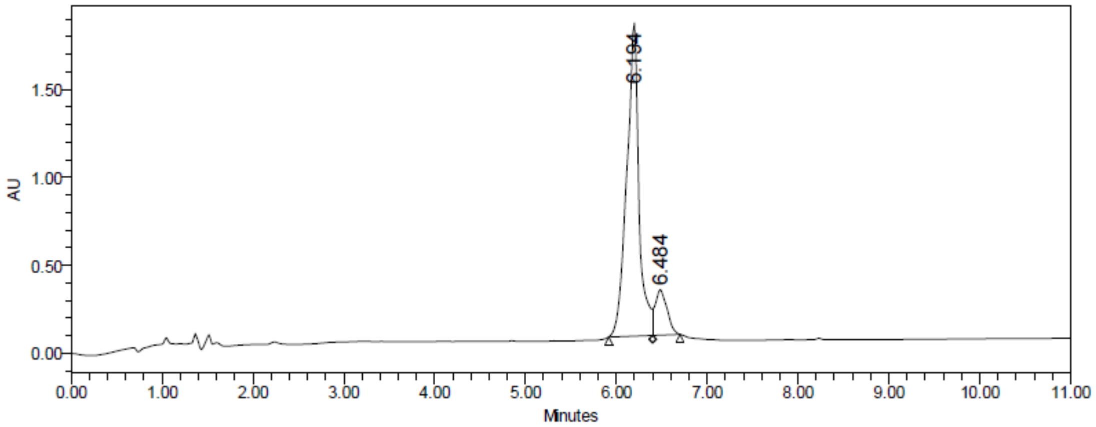

	RT	Area	% Area	Height
1	6.194	16627023	87.54	1788581
2	6.484	2365927	12.46	258551

m/z (ESI):

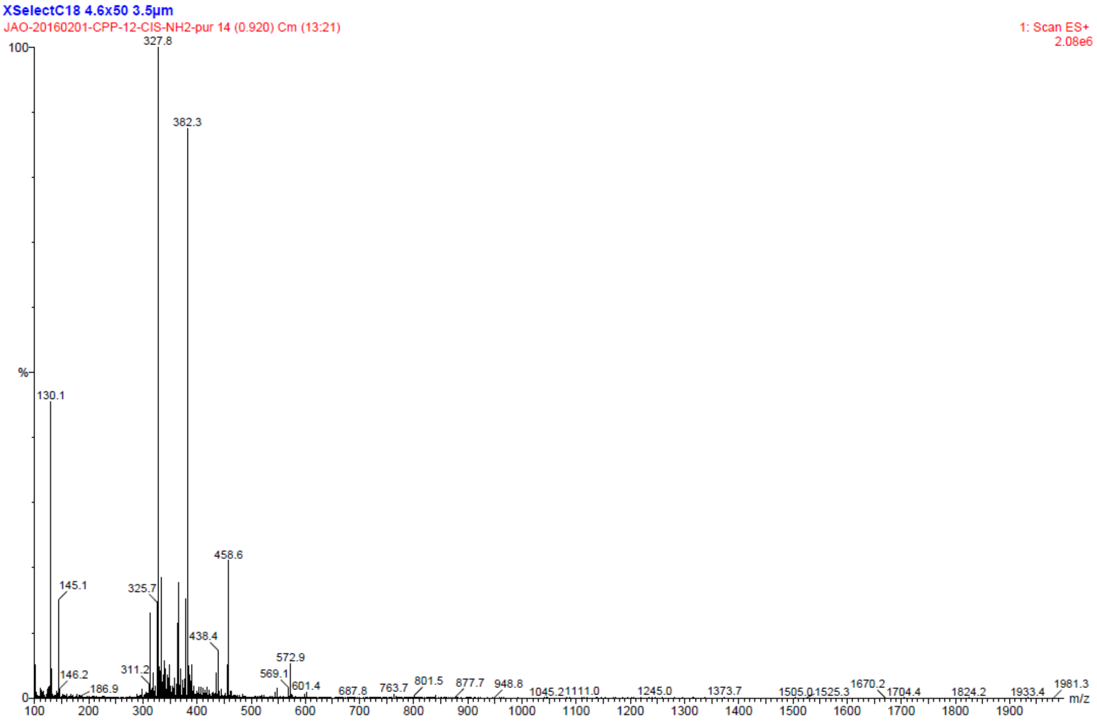

<u>Peptide γ-CC 5</u>:

RP-HPLC:

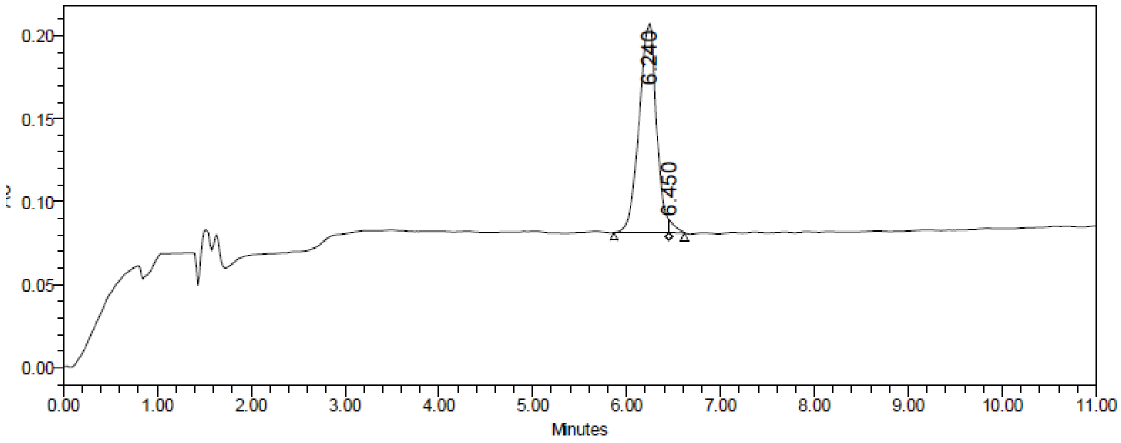

	RT	Area	% Area	Height
1	6.240	1606694	97.97	126971
2	6.450	33365	2.03	8031

m/z (ESI):

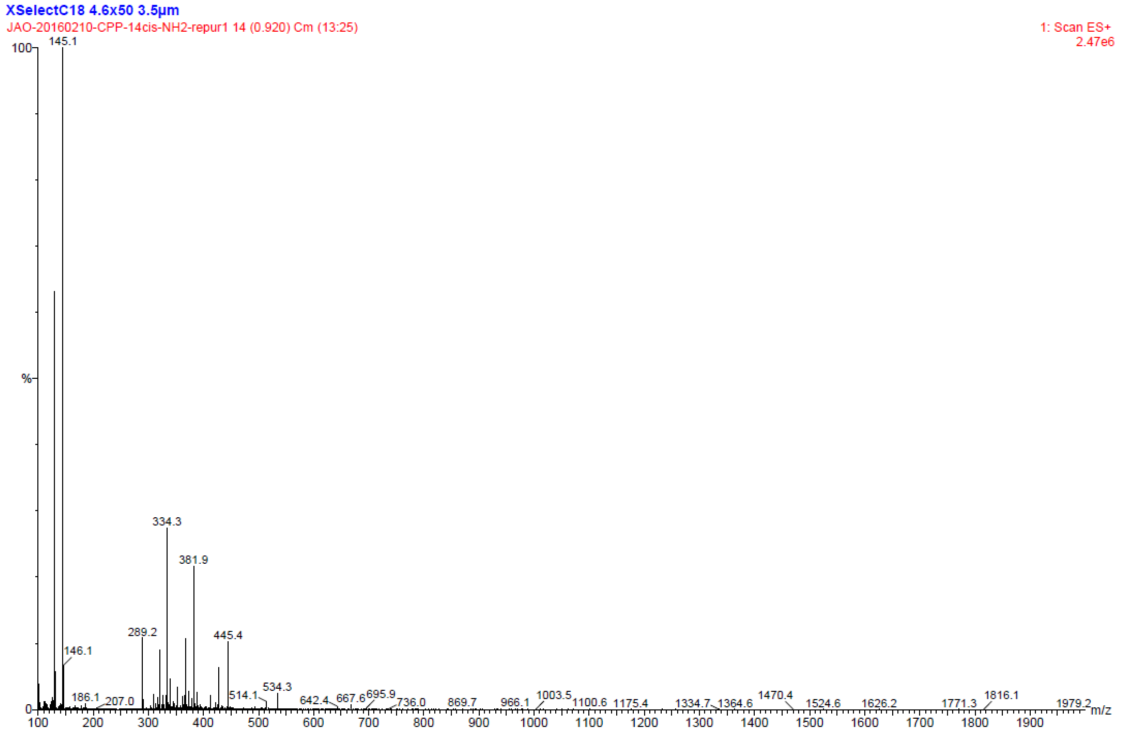

Peptide CF-γ-CC 6:

RP-HPLC:

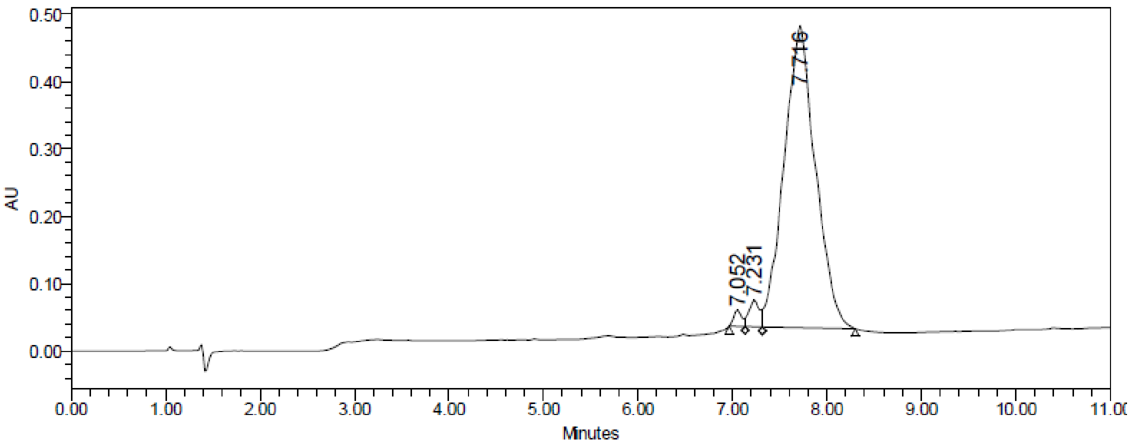

	RT	Area	% Area	Height
1	7.052	145433	1.35	23799
2	7.231	302862	2.82	38633
3	7.716	10301699	95.83	447037

m/z (ESI):

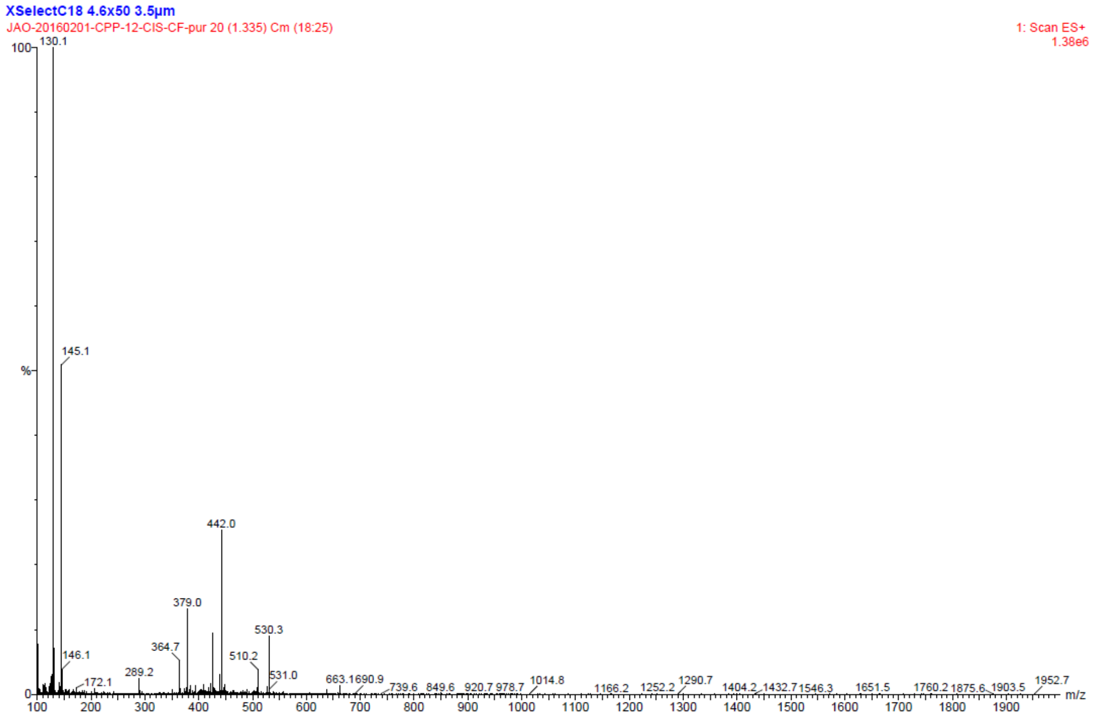

<u>Peptide CF-γ-CC 7</u>:

RP-HPLC:

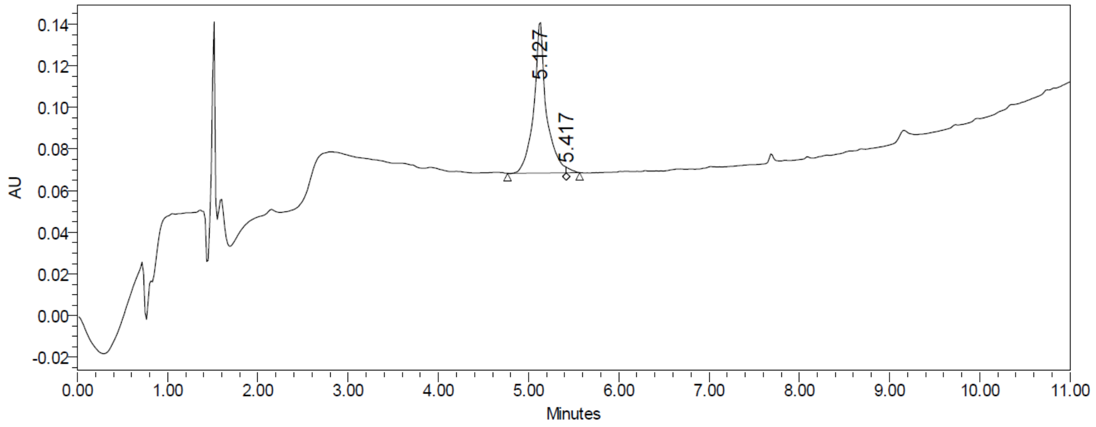

	RT	Area	% Area	Height
1	5.127	698887	98.56	72161
2	5.417	10201	1.44	2761

***m/z* (ESI):**

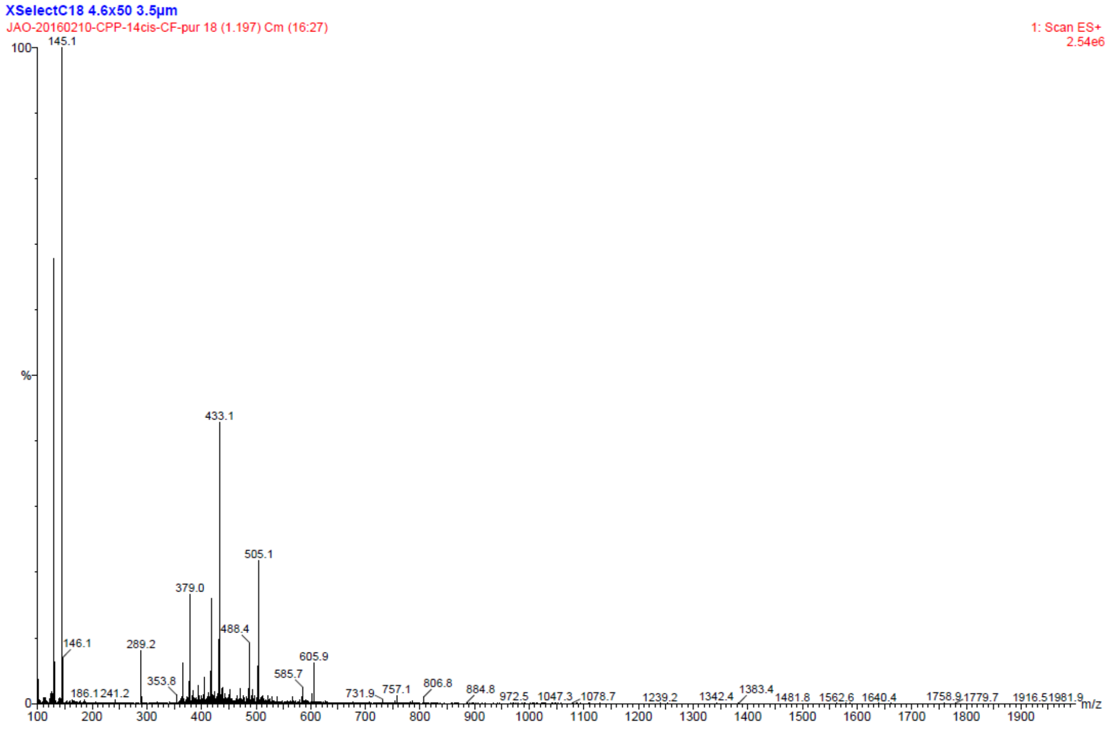

<u>Peptide γ-CT 8</u>:

RP-HPLC:

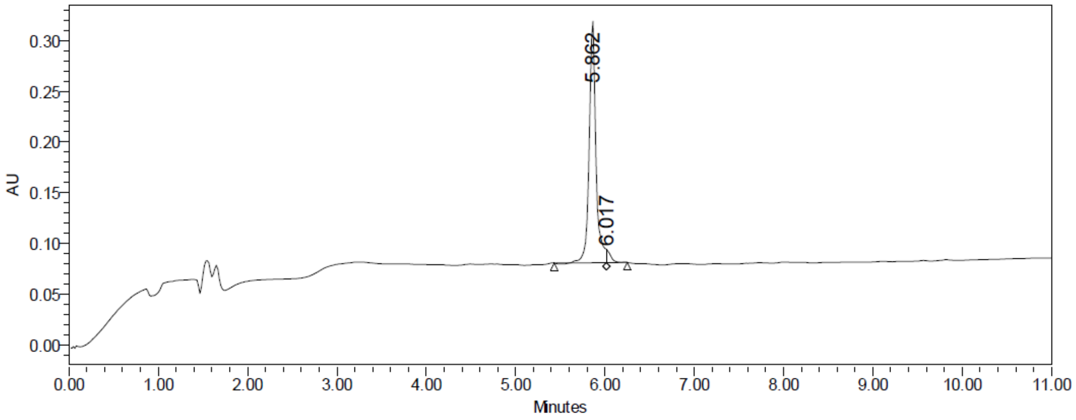

	RT	Area	% Area	Height
1	5.862	1257663	96.64	233242
2	6.017	43777	3.36	13170

***m/z* (ESI):**

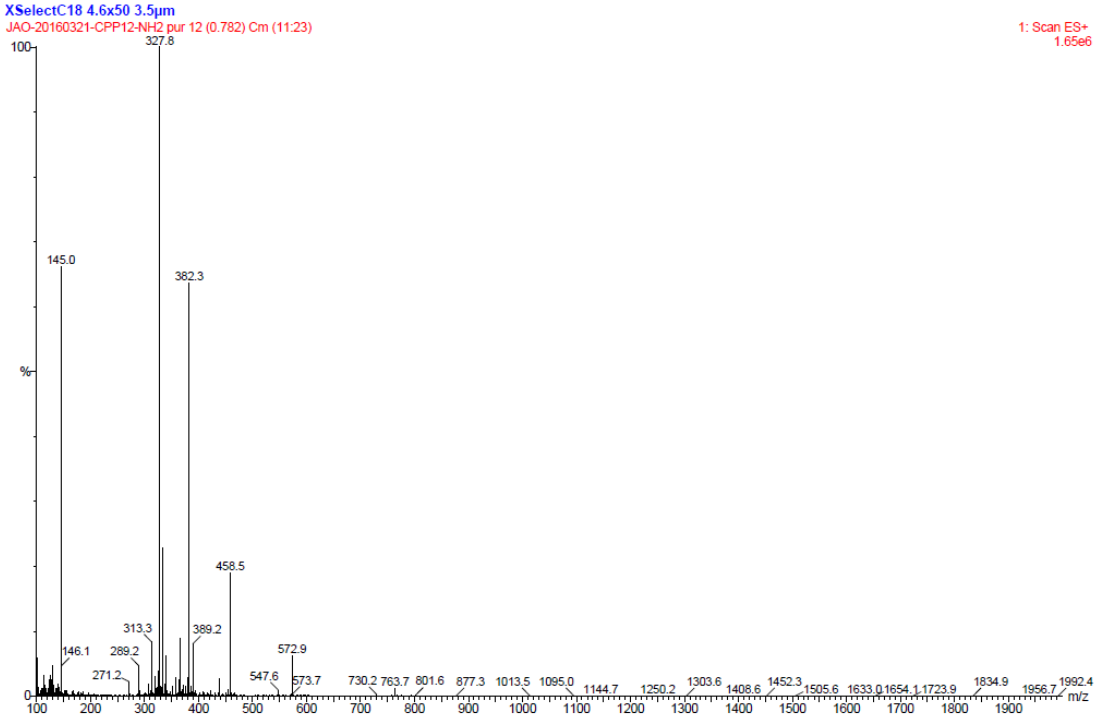

Peptide γ-CT 9:

RP-HPLC:

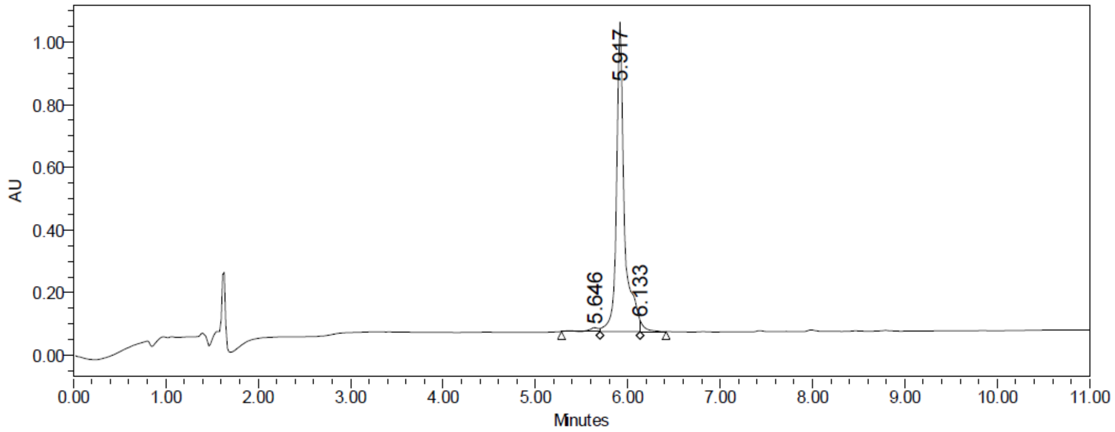

	RT	Area	% Area	Height
1	5.646	101098	1.68	12574
2	5.917	5813831	96.41	962838
3	6.133	115564	1.92	35876

***m/z* (ESI):**

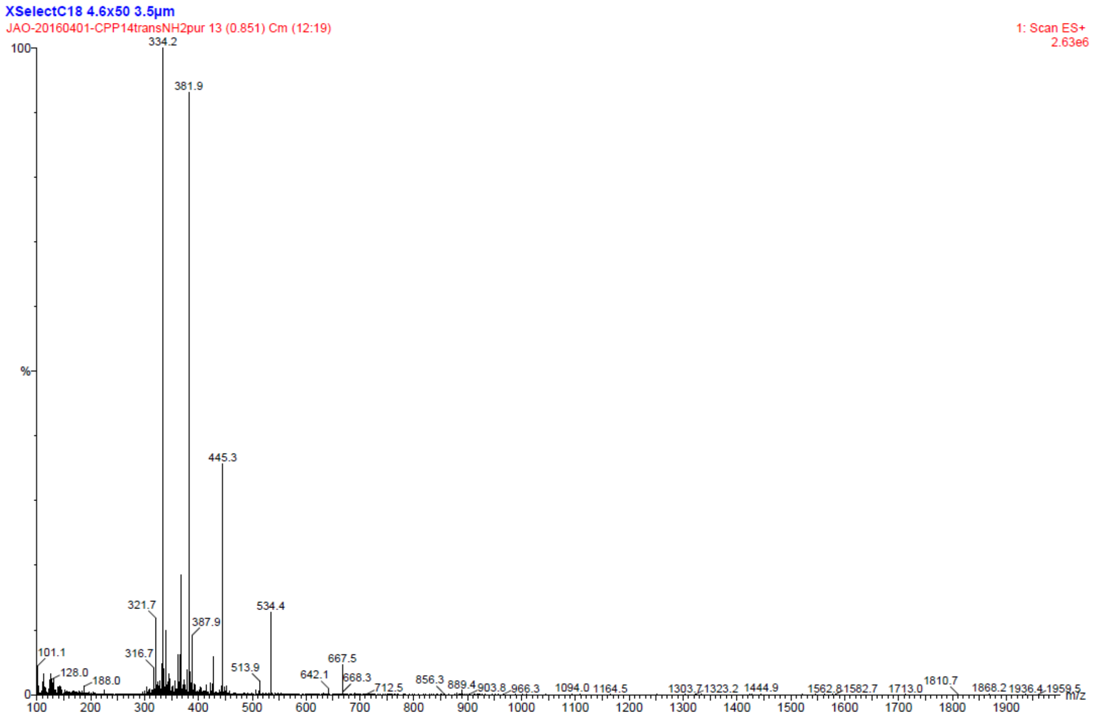

Peptide CF-γ-CT 10:

RP-HPLC:

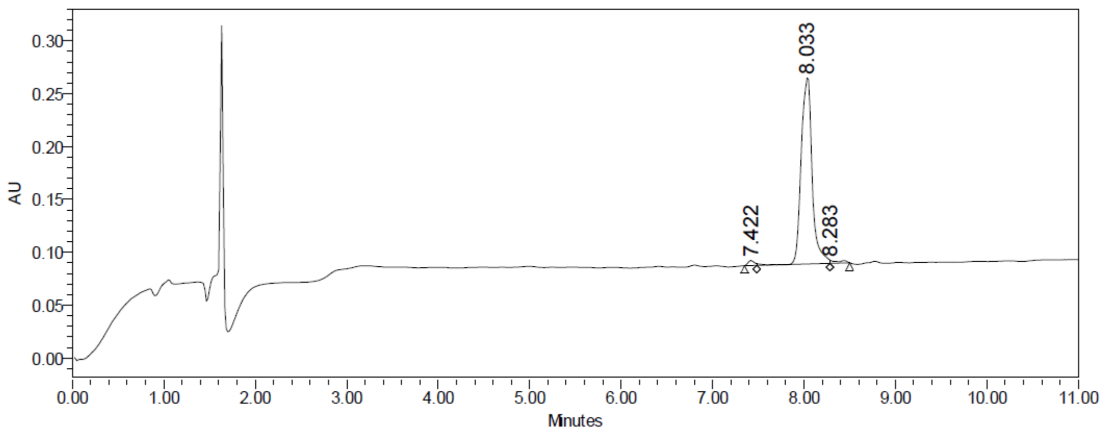

	RT	Area	% Area	Height
1	7.422	22129	1.46	4612
2	8.033	1468924	97.09	176559
3	8.283	21925	1.45	3091

m/z **(ESI):**

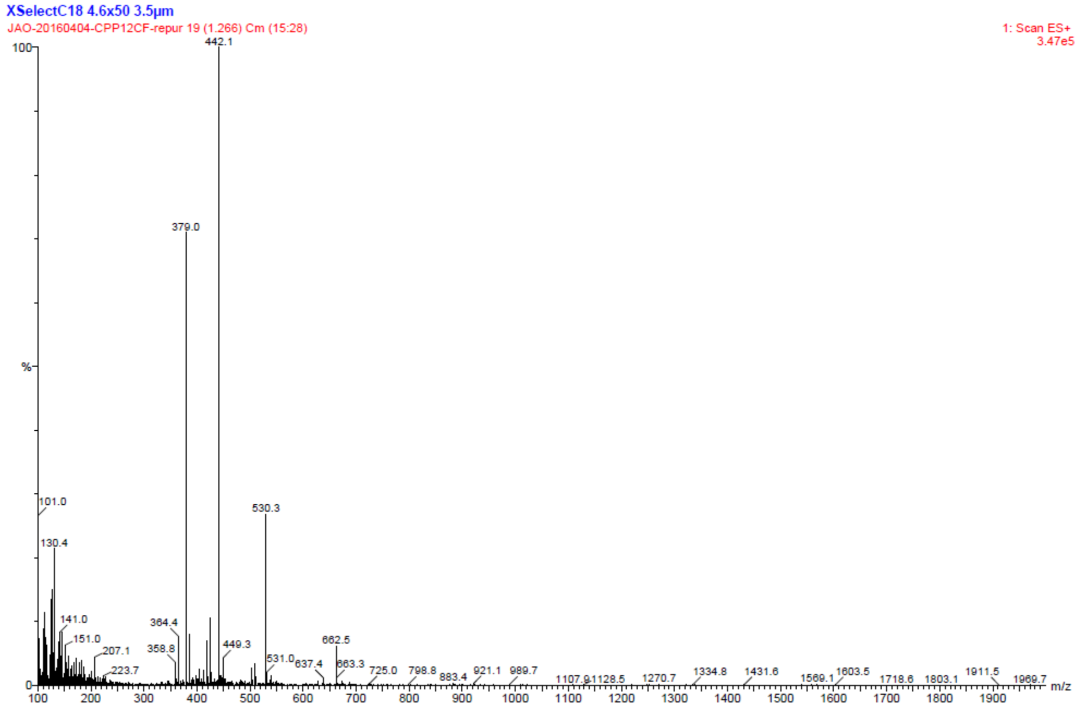

Peptide CF-γ-CT 11:

RP-HPLC:

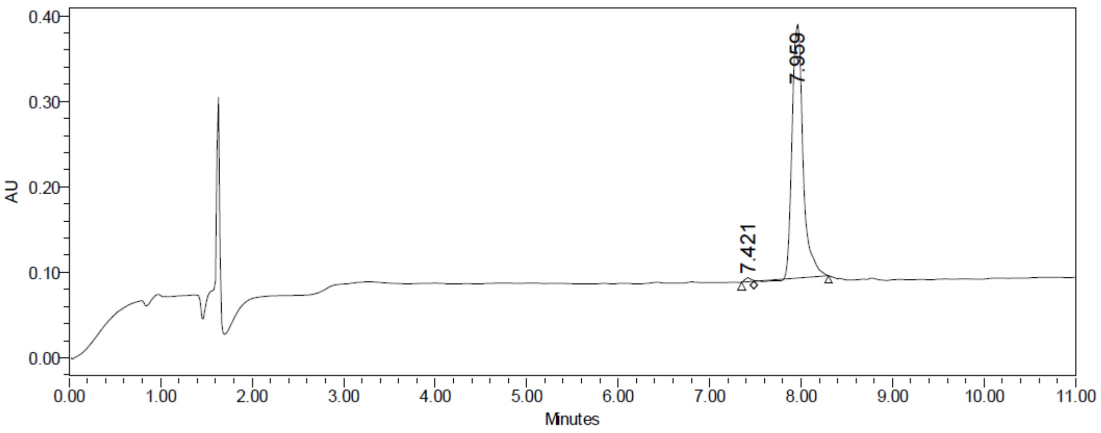

	RT	Area	% Area	Height
1	7.421	20547	0.82	4500
2	7.959	2489072	99.18	299187

m/z (ESI):

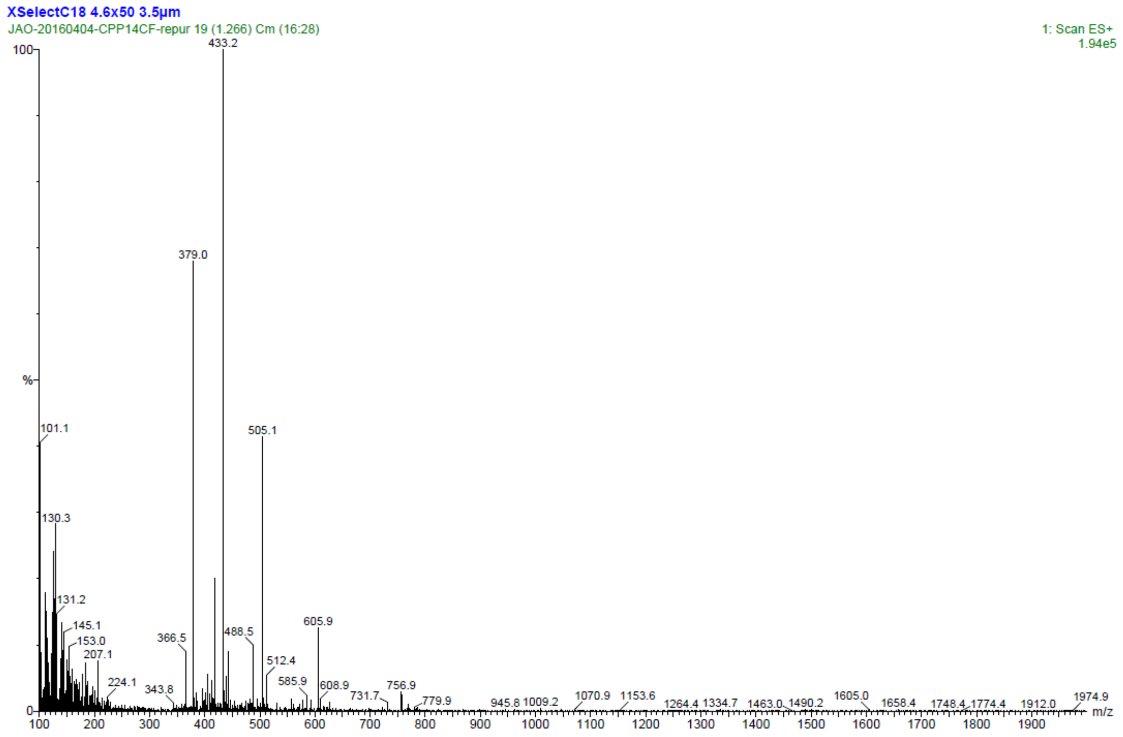

<u>Peptide 12</u>:

RP-HPLC:

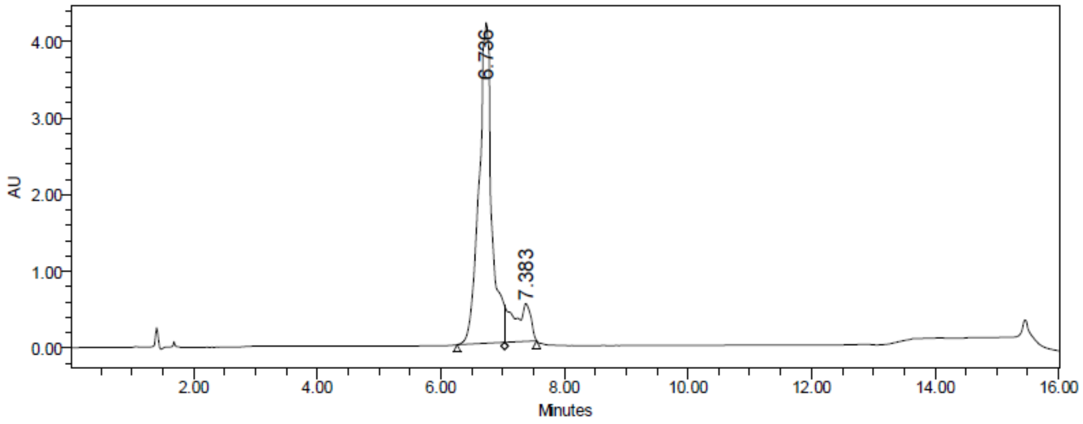

	RT	Area	% Area	Height
1	6.736	60074267	85.54	4157276
2	7.383	10151334	14.46	491368

***m/z* (ESI):**

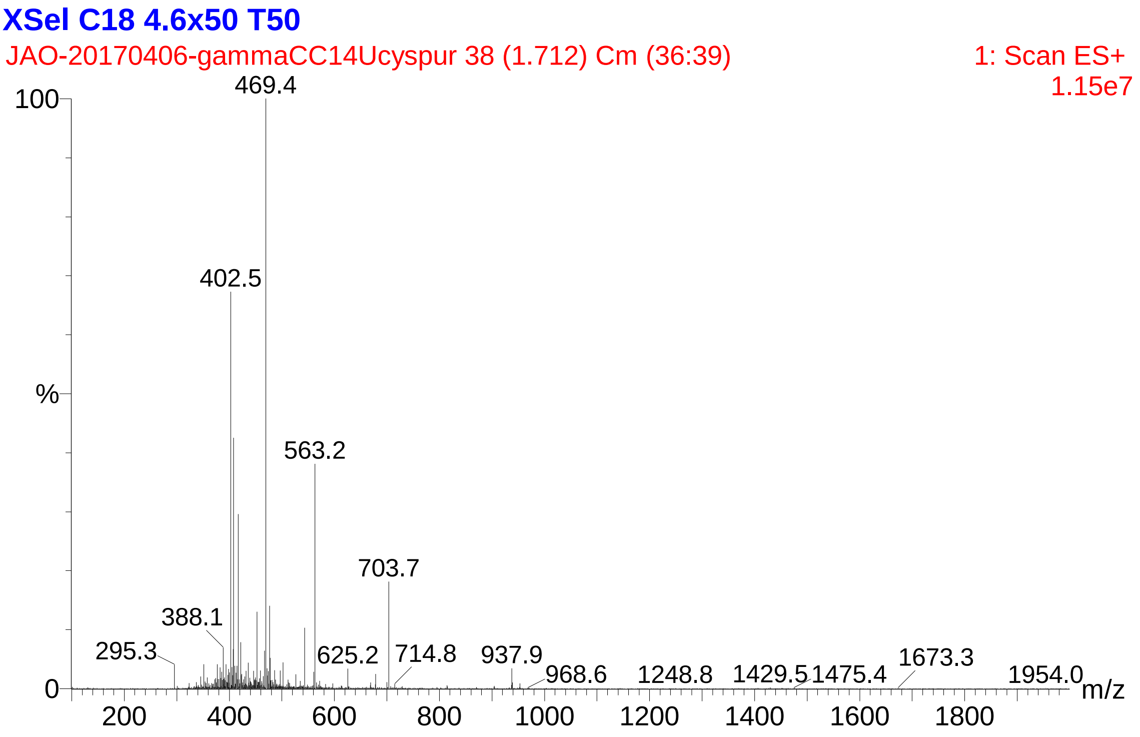

<u>Peptide 13</u>:

RP-HPLC:

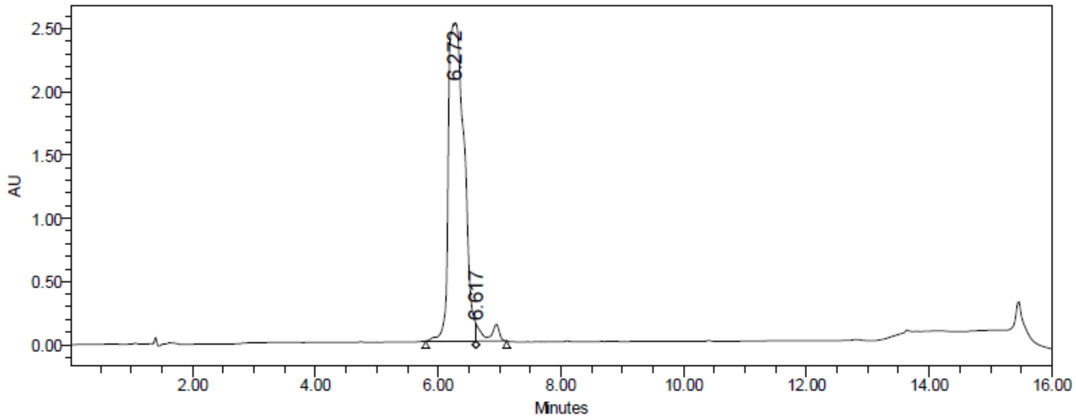

	RT	Area	% Area	Height
1	6.272	44435444	96.20	2513611
2	6.617	1755590	3.80	135637

***m/z* (ESI):**

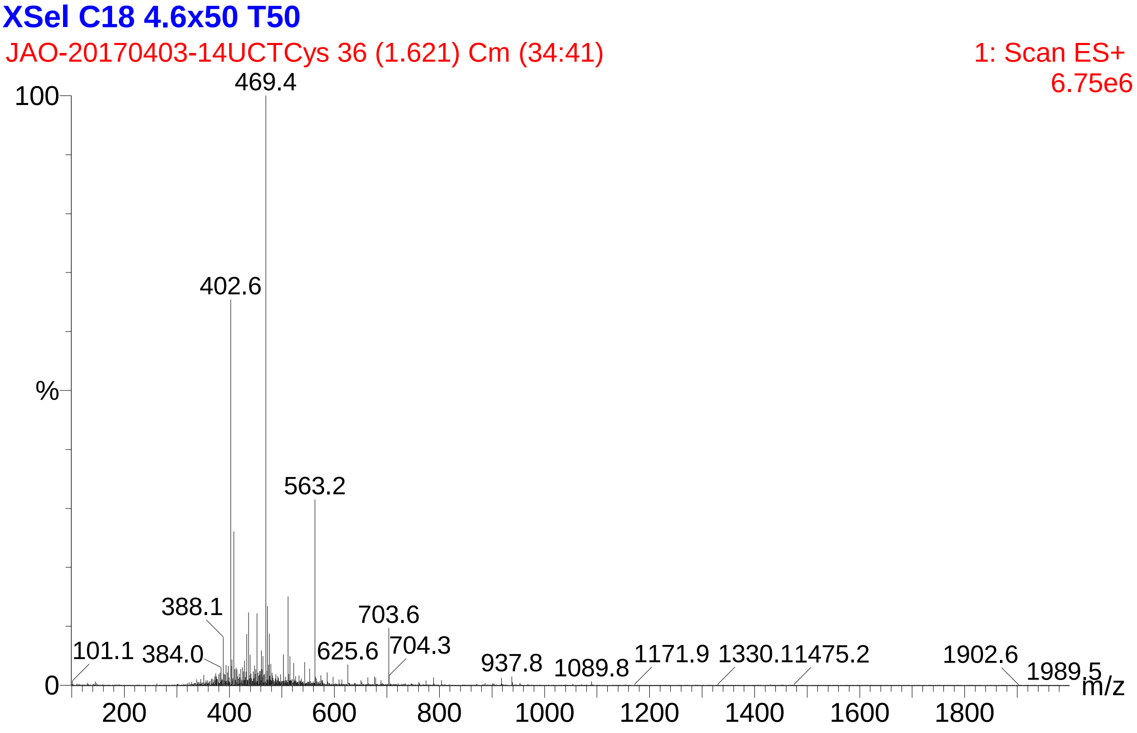

<u>Conjugate Dox-γ-CC 15</u>:

RP-HPLC:

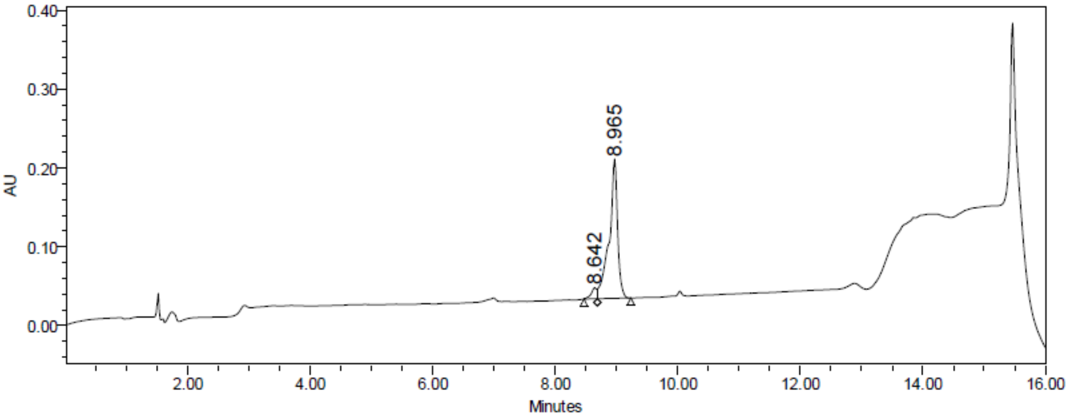

	RT	Area	% Area	Height
1	8.642	85964	4.89	13795
2	8.965	1672308	95.11	175938

m/z **(ESI):**

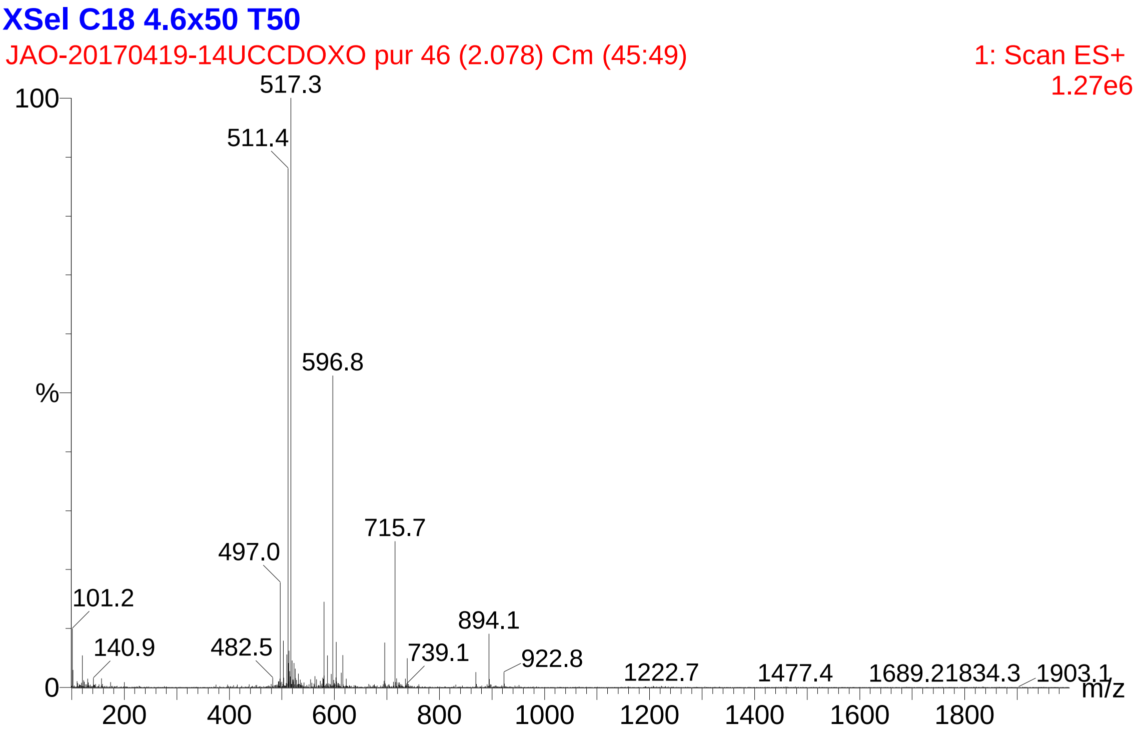

Conjugate Dox-γ-CC 16:

RP-HPLC:

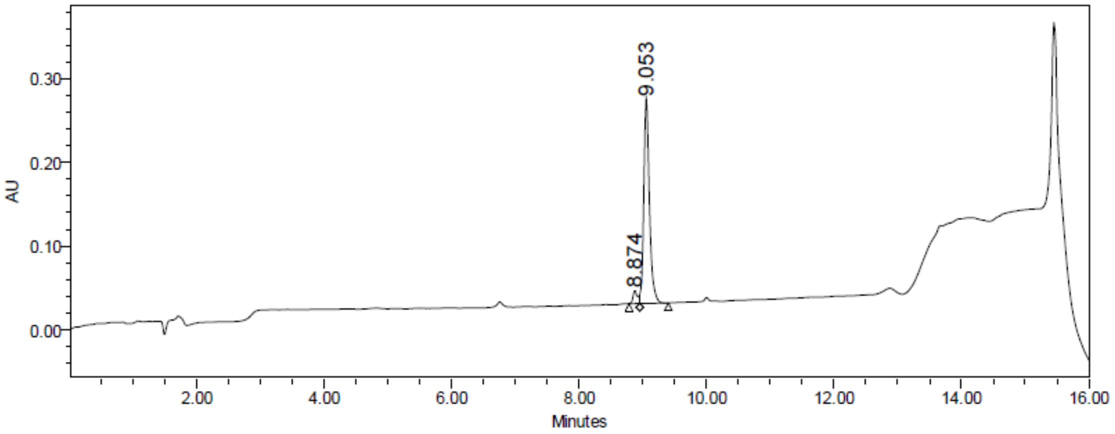

	RT	Area	% Area	Height
1	8.874	77375	4.87	14916
2	9.053	1510707	95.13	245160

***m/z* (ESI):**

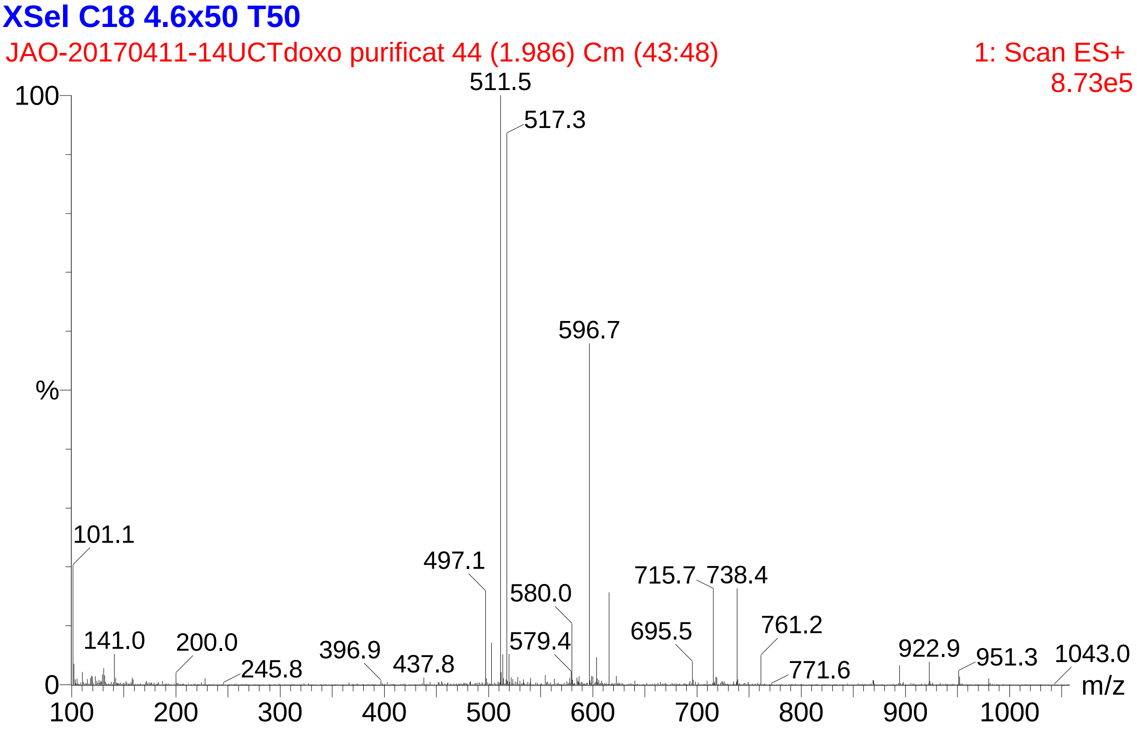

ABBREVIATIONS

ATR, Attenuated Total Reflectance; PBS, phosphate buffered saline; PyBOP, Benzotriazol-1-yl-oxytripyrrolidinophosphonium hexafluorophosphate; DIC, N,N-Diisopropylcarbodiimide; TIS, Triisopropylsilane

REFERENCES

1. Choi, S.; Isaacs, A.; Clements, D.; Liu, D.; Kim, H.; Scott, R. W.; Winkler, J. D.; DeGrado, W. F. De novo design and in vivo activity of conformationally restrained antimicrobial arylamide foldamers. *Proc. Natl. Acad. Sci. USA* **2009**, *106*, 6968-6973.

2. Sciortino, G.; Sanchez-Aparicio, J. E.; Rodríguez-Guerra Pedregal, J.; Garribba, E.; Maréchal, J. D. Computational insight into the interaction of oxaliplatin with insulin. *Metallomics* **2019**, *11*, 765-773.
